# RhoD Inhibits RhoC-ROCK-Dependent Cell Contraction via PAK6

**DOI:** 10.1016/j.devcel.2017.04.010

**Published:** 2017-05-08

**Authors:** Charlotte H. Durkin, Flavia Leite, João V. Cordeiro, Yutaka Handa, Yoshiki Arakawa, Ferran Valderrama, Michael Way

**Affiliations:** 1 Cellular Signalling and Cytoskeletal Function Laboratory, The Francis Crick Institute, 1 Midland Road, London NW1 1AT, UK

**Keywords:** RhoC, RhoD, RhoGTPase crosstalk, cell contraction, blebbing, Pak6, Vaccinia virus

## Abstract

RhoA-mediated regulation of myosin-II activity in the actin cortex controls the ability of cells to contract and bleb during a variety of cellular processes, including cell migration and division. Cell contraction and blebbing also frequently occur as part of the cytopathic effect seen during many different viral infections. We now demonstrate that the vaccinia virus protein F11, which localizes to the plasma membrane, is required for ROCK-mediated cell contraction from 2 hr post infection. Curiously, F11-induced cell contraction is dependent on RhoC and not RhoA signaling to ROCK. Moreover, RhoC-driven cell contraction depends on the upstream inhibition of RhoD signaling by F11. This inhibition prevents RhoD from regulating its downstream effector Pak6, alleviating the suppression of RhoC by the kinase. Our observations with vaccinia have now demonstrated that RhoD recruits Pak6 to the plasma membrane to antagonize RhoC signaling during cell contraction and blebbing.

## Highlights

• Vaccinia F11 protein is required for virus-induced cell contraction and blebbing
• F11-induced cell contraction depends on RhoC, but not RhoA, signaling to ROCK
• RhoD recruits Pak6 to the plasma membrane to antagonize RhoC signaling
• F11 inhibits RhoD signaling to its downstream effector Pak6

## Graphical Abstract

**Figure:**
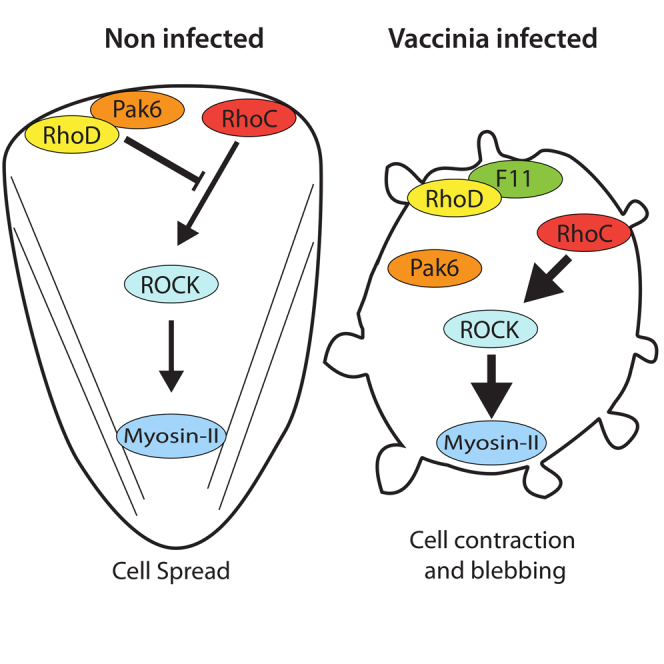

## Teaser

Many different viral infections induce cell contraction and blebbing. Durkin, Leite, et al. show that during vaccinia infection, RhoC and not RhoA regulates this ROCK-mediated cytopathic effect of cell contraction. They delineate a pathway for RhoC regulation, in which the viral protein F11 blocks RhoC antagonism by RhoD-recruited Pak6.

## Introduction

The cortical actin cytoskeleton consists of a dense network of actin filaments, crosslinked by myosin-II and other actin-binding proteins, that is intrinsically linked to the cytoplasmic face of the plasma membrane (Biro et al., 2013, Charras et al., 2006, Clark et al., 2013, Fritzsche et al., 2013, Koster and Mayor, 2016, Morone et al., 2006). The regulation of the density and organization of actin filaments in the cell cortex, as well as their myosin-II driven contraction, provides the cell with mechanical resilience (Bovellan et al., 2014, Clark et al., 2013, Fritzsche et al., 2013, Fritzsche et al., 2016, Gauthier et al., 2012, Salbreux et al., 2012, Tinevez et al., 2009). It also enables the cortical actin cytoskeleton to regulate the shape of the cell during a variety of cellular processes including migration, mitotic rounding, and cytokinesis (Friedl and Wolf, 2003, Matthews et al., 2012, Sedzinski et al., 2011, Zatulovskiy et al., 2014). A frequent hallmark of myosin-II-driven contraction of the actin cortex during these cellular processes is the transient and rapid appearance of spherical blebs on the plasma membrane (Charras et al., 2008). These protrusions occur at regions where the plasma membrane separates from the underlying actin cortex because of increased hydrostatic pressure within the cell (Charras et al., 2008, Paluch et al., 2006, Tinevez et al., 2009).

Over the last decade, it has become clear that blebbing of the plasma membrane helps drive ameboid-based cell motility during development and tumor cell migration (Blaser et al., 2006, Charras and Paluch, 2008, Diz-Munoz et al., 2010, Fackler and Grosse, 2008, Friedl and Wolf, 2003, Kardash et al., 2010, Paluch and Raz, 2013, Sahai and Marshall, 2003, Sanz-Moreno et al., 2008, Tozluoglu et al., 2013). A key determinant for assembly and contraction of the actin cortex is the activation of myosin-II by ROCK-mediated phosphorylation of the myosin light chain (MLC) (Amano et al., 1997, Amano et al., 1996, Amano et al., 2010, Amin et al., 2013, Sahai and Marshall, 2003). ROCK also indirectly increases the activity of myosin-II by inhibiting the MLC phosphatase through phosphorylation of the myosin phosphatase-targeting subunit 1 (MYPT1) (Amano et al., 2010, Amin et al., 2013, Kawano et al., 1999). The small guanosine triphosphatase (GTPase) RhoA is most widely implicated in activating ROCK to drive cell contraction (Charras et al., 2006, Costigliola et al., 2010, Gutjahr et al., 2005, Sahai and Marshall, 2003, Sanz-Moreno et al., 2008). However, ROCK can also interact with the closely related GTPase RhoC, which is known to promote ameboid-based motility, tumor invasion, and metastasis (Clark et al., 2000, Hakem et al., 2005, Kitzing et al., 2010, Leung et al., 1996, Ruth et al., 2006, Sahai and Marshall, 2002, Simpson et al., 2004).

Cell contraction and membrane blebbing are also frequently observed as part of the cytopathic effect induced by many different viruses during their replication cycles (Agol, 2012). For example, within a few hours of vaccinia virus infection, cells begin to contract and bleb, in a process that is independent of apoptosis (Bablanian et al., 1978, Barry et al., 2015, Schepis et al., 2006, Schramm et al., 2006). Previous observations suggest the vaccinia protein F11, which is expressed early in infection, is involved in vaccinia-induced cell contraction and/or the loss of cell-cell adhesion (Cordeiro et al., 2009, Morales et al., 2008, Valderrama et al., 2006). F11 downregulates RhoA signaling in the latter stages of the virus replication cycle (Arakawa et al., 2007b, Cordeiro et al., 2009, Valderrama et al., 2006). F11 binds directly to GTP-bound RhoA using a motif that is conserved in ROCK (Cordeiro et al., 2009, Valderrama et al., 2006). Once bound, RhoA is inactivated by the Rho GTPase-activating protein (RhoGAP) activity of myosin-9A, which binds to the central PDZ-like domain of F11 via its C-terminal PDZ-binding motif (Handa et al., 2013). Ultimately, F11-mediated inhibition of RhoA signaling late during viral replication promotes the spread of vaccinia infection by stimulating cell migration, increasing microtubule dynamics, and enhancing viral release by modulating the cortical actin beneath the plasma membrane (Arakawa et al., 2007a, Arakawa et al., 2007b, Cordeiro et al., 2009, Handa et al., 2013, Valderrama et al., 2006). Consistent with its role during vaccinia virus infection, ectopic expression of F11 can enhance the cell-to-cell spread and oncolytic potential of myxoma virus, which lacks F11 (Irwin and Evans, 2012, Irwin et al., 2013).

Previous studies on F11 have largely focused on its role in infected cells at 8–20 hr post infection (hpi) (Arakawa et al., 2007a, Arakawa et al., 2007b, Cordeiro et al., 2009, Handa et al., 2013, Valderrama et al., 2006). However, F11 expression correlates with the onset of virus-induced cell contraction (Cordeiro et al., 2009, Morales et al., 2008). With this in mind, we set out to investigate whether and how F11 plays a role in vaccinia-induced cell contraction and blebbing. We found that vaccinia stimulates cell contraction independently of RhoA by activating RhoC-mediated signaling to ROCK. Furthermore, the ability of RhoC to promote this effect depends on F11-mediated inhibition of RhoD signaling to Pak6.

## Results

### F11 Induces Cell Contraction Early during Vaccinia Infection

**Figure 1:**
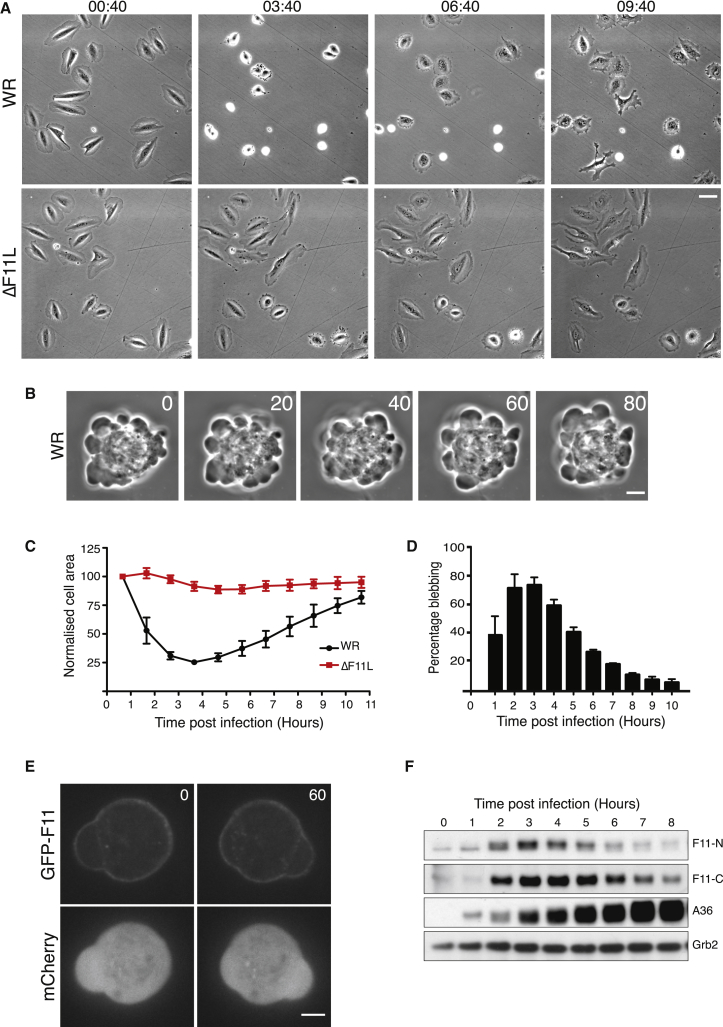
Vaccinia Stimulates F11-Dependent Cell Contraction (A) Phase-contrast images showing the morphology of HeLa cells at the indicated time points (hours:minutes) after infection with WR or the ΔF11L virus (see Movie S1). Scale bar, 30 μm. (B) Phase-contrast images, 20 s apart, showing WR-induced bleb formation in HeLa cells 3 hr 40 min after infection. Scale bar, 5 μm. (C) Quantification of the average area of HeLa cells infected with WR (black) or ΔF11L (red) over 11 hr. Each cell area is normalized to its initial value at the start of acquisition. (D) Quantification of the percentage of WR-infected cells blebbing at the indicated times post infection. (E) Images showing the association of GFP-F11 with the plasma membrane in cells infected with WR for 3 hr 40 min. The time is indicated in seconds and mCherry provides a volume marker (see Movie S2). Scale bar, 5 μm. (F) Immunoblot analysis with two different F11 antibodies reveals the level of endogenous F11 expression in HeLa cells at the indicated times after infection with WR. A36 and Grb2 represent viral and HeLa cell loading controls, respectively. Error bars in graphs represent the SEM from three independent experiments, in which a total of 60 cells were analyzed. See also Figure S1.

In agreement with earlier studies, live-cell imaging demonstrates that within the first few hours of infection the Western Reserve (WR) strain of vaccinia induces contraction and blebbing of HeLa cells (Figures 1A and 1B; Movie S1). Quantification of the cell area reveals that maximum contraction and blebbing occurs approximately 3 hr 40 min post infection, after which time infected cells begin to respread (Figures 1C and 1D). Vaccinia-induced cell contraction is not specific to HeLa cells, as it is also seen in U-2 OS cells (Figure S1A). In contrast, infection with WR lacking the F11L gene (ΔF11L virus) does not induce cells to contract or bleb (Figures 1A, 1C, and S1A). In agreement with Schepis et al. (2006), we also found that the highly attenuated virus strain, Modified Vaccinia Ankara (MVA), which does not express a functional F11, did not induce HeLa cell contraction early during infection (Figure S1B). Using a recombinant virus expressing GFP-tagged F11 from its endogenous promoter, we found that F11 associates with the plasma membrane, consistent with a possible role in regulating the actin cortex (Figure 1E and Movie S2). Immunoblot analysis of WR-infected cells confirmed that the onset of cell contraction correlates with the expression of F11 (Figure 1F). Moreover, respreading correlates with a gradual loss of F11, suggesting that its presence promotes vaccinia-induced cell contraction. Consistent with this, prolonged expression of F11 in U-2 OS cells suppresses their respreading at later time points (Figure S1A). A role for F11 in promoting cell contraction is unexpected, given previous observations demonstrating that the viral protein inhibits, rather than activates, RhoA signaling.

### Vaccinia-Induced Cell Contraction Depends on ROCK

**Figure 2:**
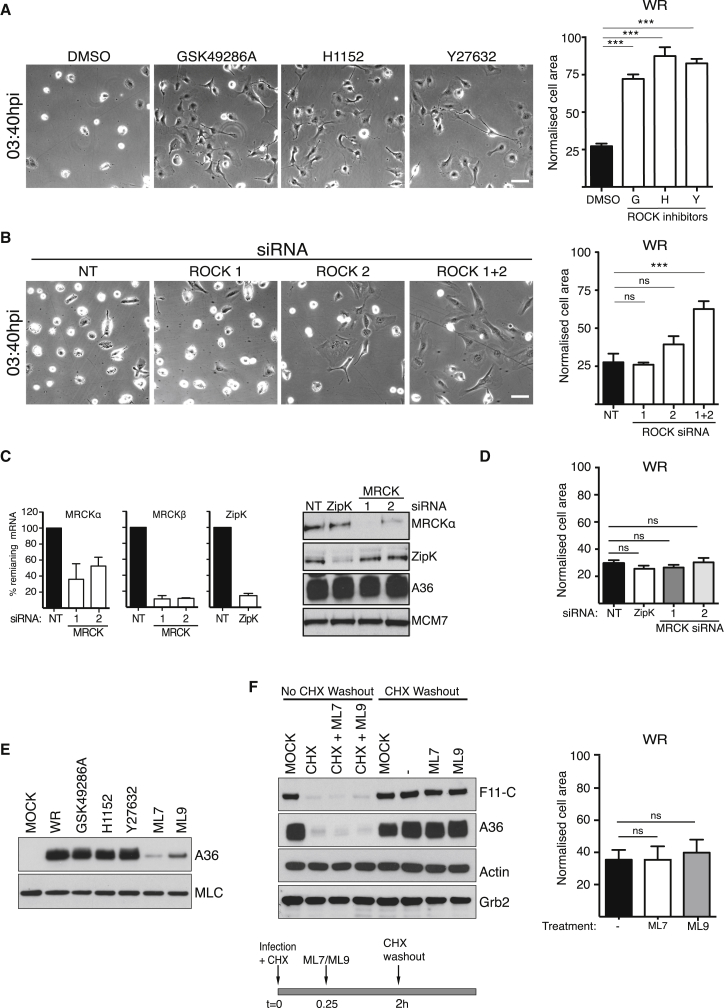
Vaccinia-Induced Cell Contraction Is Dependent on ROCK Signaling (A) Images of HeLa cells infected with WR for 3 hr 40 min in the absence (DMSO) or presence of ROCK inhibitors (GSK49286A, H1152, and Y27632) together with quantification of cell area. Scale bar, 30 μm. (B) Images showing the morphology of HeLa cells treated with siRNA against ROCK1 and ROCK2 at 3 hr 40 min post infection with WR together with quantification of cell area. Scale bar, 30 μm. (C) The graphs show the percentage of remaining MRCKα, MRCKβ, or ZipK mRNA following treatment with the indicated siRNA relative to the non-targeting AllStar control (NT). The Immunoblot shows the level of MRCKα and ZipK after siRNA treatment. A36 and MCM7 represent viral and HeLa cell loading controls, respectively. (D) Quantification of the average area of HeLa cells infected with WR for 3 hr 40 min and treated with the indicated siRNA. (E) Immunoblot analysis at 5 hpi reveals that in contrast to the ROCK inhibitors, ML7 and ML9 suppress viral entry based on reduced early viral protein expression (A36). MLC represents a cell loading control. (F) Immunoblot analysis demonstrates that washout of cycloheximide at 2 hpi in the presence or absence of ML7 and ML9 does not inhibit viral entry based on expression of F11 and A36. Actin and Grb2 represent loading controls. The graph reveals that after cycloheximide washout WR-infected cells still contract in the presence of ML7 and ML9. Error bars in graphs represent the SEM from three independent experiments in which a total of 60–100 cells were analyzed. ^∗∗∗^p < 0.001; ns, not significant. See also Figure S2.

It is well established that RhoA signaling to ROCK promotes myosin-II-mediated cell contraction and blebbing by phosphorylating MLC and also inhibiting MYPT1 (Amin et al., 2013, Julian and Olson, 2014). To investigate whether ROCK is also required for vaccinia-induced cell contraction, we infected cells in the presence of one of three different ROCK inhibitors. In all cases, we found that there was a dramatic inhibition in virus-induced cell contraction, consistent with the reduction in MLC2 and MYPT1 phosphorylation seen in immunoblots (Figures 2A and S2A). Lack of cell contraction was not due to an inhibition of viral entry, as the drug treated cells were equally as well infected as control cells (Figure S2B). To extend our pharmacological analysis, we examined the impact of small interfering RNA (siRNA)-mediated depletion of ROCK1 and ROCK2 on virus-induced cell contraction. Independent knockdown of either kinase did not significantly impair cell contraction (Figures 2B and S2C). When both proteins were depleted, early viral protein expression was not impaired but there was a substantial inhibition of cell contraction (Figures 2B and S2D). To explore whether additional signaling pathways also contribute to myosin-II activation, we treated cells with siRNA against myotonic dystrophy kinase-related Cdc42-binding kinases (MRCKα/β) and zipper-interacting protein kinase (ZIPK) (Nehru et al., 2013, Usui et al., 2014, Zhao and Manser, 2015). We found that WR-infected cells still contract in the absence of MRCKα/β and ZIPK (Figures 2C and 2D). In addition, we treated infected cells with ML7 or ML9 to inhibit MLC kinase (MLCK) to examine whether it also participates in virus-driven cell contraction. Consistent with a role for myosin-II during viral entry (Mercer and Helenius, 2008), we found that inhibition of MLCK with ML7 or ML9 dramatically reduced early viral protein expression (Figure 2E). To overcome this inhibition, we infected cells in the presence of cycloheximide to block viral uncoating (Mercer et al., 2012), before adding ML7 or ML9 (Figure 2F). Removal of the cycloheximide in the presence of ML7 or ML9 restarts the stalled replication cycle, resulting in early viral protein expression and cell contraction (Figure 2F). Taken together, our results indicate that ROCK-mediated regulation of myosin-II is required for vaccinia-induced cell contraction and that both isoforms are functionally redundant. The global levels of phospho-MLC did not appreciably change during WR or ΔF11L virus infection, but the subcellular distribution of phospho-MLC was strikingly different (Figures S2A and S2E). Live-cell imaging also reveals that RFP-MLC2 is recruited to the blebbing cell cortex (Figure S2F and Movie S3), suggesting that localized ROCK activity drives the redistribution of myosin-II to promote cell contraction.

### Vaccinia Promotes RhoC- but Not RhoA-Dependent Cell Contraction

**Figure 3:**
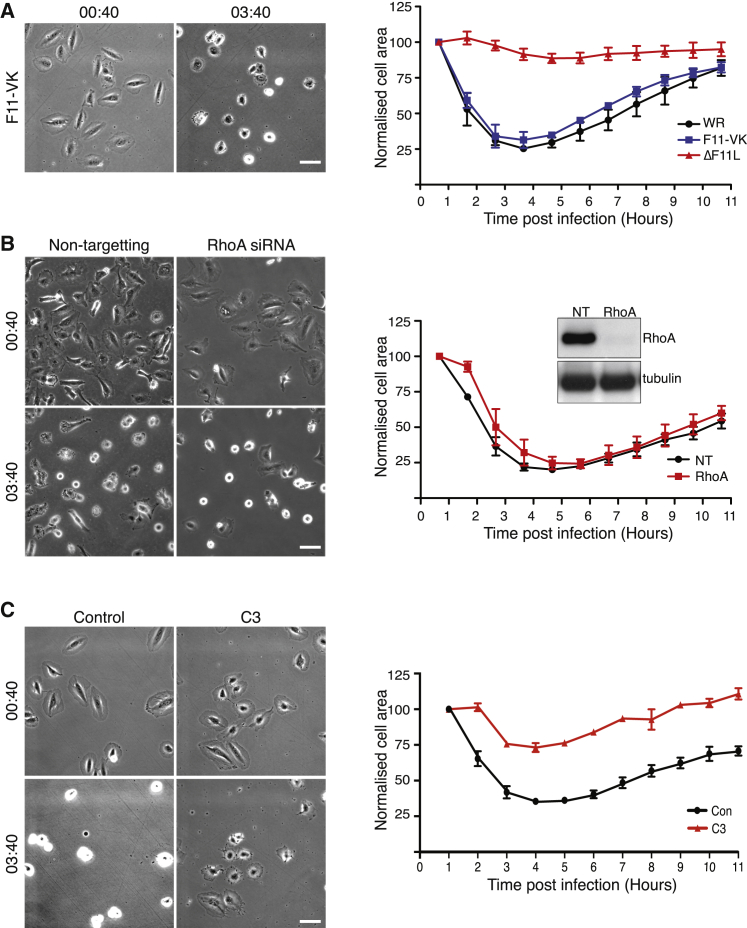
Vaccinia-Mediated Cell Contraction Is Not Dependent on RhoA (A) Phase-contrast images of HeLa cells infected with the F11-VK virus and quantification of their average area (blue line). The F11-VK infection was performed at the same time as WR (black line) and ΔF11L (red line) taken from Figure 1A. (B) Images of HeLa cells infected with WR treated with non-targeting (NT) control or RhoA siRNA, together with quantification of their average area. The immunoblot shows the efficiency of RhoA knockdown, and α-tubulin represents a loading control. (C) Representative images of HeLa cells infected with WR in the presence or absence of the C3 Rho inhibitor, together with quantification of their average area. All scale bars, 5 μm. Error bars represent the SEM from three independent experiments, in which a total of 60 (A), 80 (B), and 100 (C) cells were analyzed. See also Figure S3.

Given the involvement of ROCK1/2, we wondered whether an interaction of F11 with RhoA is also required for virus-induced cell contraction. To address this question, we infected cells with a recombinant virus expressing F11-VK, which is defective in RhoA binding (Cordeiro et al., 2009). We found that the F11-VK virus is as effective as the parental WR strain in inducing cell contraction (Figure 3A and Movie S1). Furthermore, siRNA-mediated depletion of RhoA did not impact on the ability of WR to induce cell contraction (Figure 3B). In contrast, the *Clostridium botulinum* toxin C3, a pan-RhoA, B, and C inhibitor (Aktories, 2011), blocked virus-induced cell contraction but not infection (Figures 3C and S3). This suggests that vaccinia-induced cell contraction is independent of RhoA but reliant on the activation of ROCK by another RhoGTPase.

**Figure 4:**
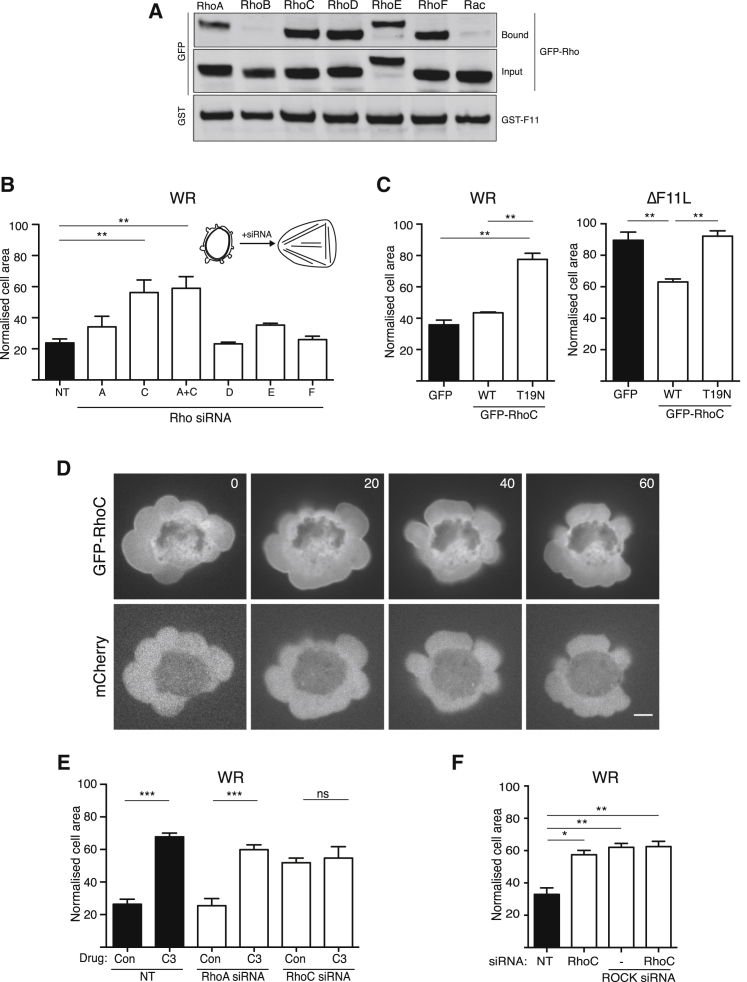
RhoC Is Required for Vaccinia-Induced Cell Contraction (A) Immunoblot analysis of glutathione pull-downs on WR-infected HeLa cell lysates reveals that GST-F11 interacts with GFP-tagged RhoA, RhoC, RhoD, RhoE, and RhoF. (B) Quantification of the area of HeLa cells treated with the indicated siRNA at 3 hr 40 min post infection with WR. (C) Quantification of the area of HeLa cells expressing GFP or GFP-tagged wild-type (WT) or T19N (dominant-negative) RhoC 3 hr 40 min after infection with WR (left) or ΔF11L (right). (D) Images showing the association of GFP-RhoC with the plasma membrane in cells infected with WR for 3 hr 40 min. The time is indicated in seconds and mCherry provides a volume marker (see Movie S4). Scale bar, 5 μm. (E) Quantification of WR-infected HeLa cell area at 3 hr 40 min post infection in the presence or absence of C3, 72 hr after treatment with the indicated siRNA. (F) Normalized cell area of WR-infected HeLa cells at 3 hr 40 min post infection, 72 hr after treatment with the indicated siRNA. Error bars in graphs represent the SEM from three independent experiments, in which a total of 90 (B), 60 (C), and 80 (E and F) cells were analyzed. ^∗^p < 0.05, ^∗∗^p < 0.01, ^∗∗∗^p < 0.001; ns, not significant. See also Figure S4.

Previous studies have linked RhoB, RhoC, RhoD, RhoE, and RhoF (Rif) to ROCK signaling (Fan et al., 2010, Leung et al., 1996, Riento and Ridley, 2003, Tsubakimoto et al., 1999). Given this finding, we examined whether F11 also interacts with these RhoGTPases. Pull-down assays on lysates from infected HeLa cells demonstrate that glutathione S-transferase (GST)-F11 associates with GFP-tagged RhoC, D, E, and F but not RhoB or Rac (Figure 4A). To examine whether any of these RhoGTPases are required for vaccinia-induced cell contraction, we performed siRNA-mediated ablation of each protein (Figures S4A and S4B). We found that only depletion of RhoC had a significant impact on the ability of WR to induce cell contraction (Figure 4B). Consistent with the lack of involvement of RhoA, the combined loss of RhoA and RhoC did not result in a greater inhibition of WR-induced cell contraction than RhoC alone (Figures 4B and S4A). In addition, expression of GFP-tagged dominant-negative RhoC-T19N blocked WR-mediated cell contraction (Figure 4C). In contrast, expression of GFP-RhoC stimulated partial contraction of ΔF11L virus-infected cells (Figure 4C). In agreement with its role in promoting cell contraction, RhoC is associated with the plasma membrane of blebs (Figure 4D and Movie S4). The involvement of RhoC explains why C3 toxin blocks WR-induced cell contraction (Figure 3C). Moreover, C3 treatment did not increase the suppression of cell contraction induced by the loss of RhoC (Figure 4E). To confirm that RhoC acts via ROCK to promote contraction of infected cells, we depleted both RhoC and ROCK1/2. Depletion of all three proteins impaired WR-induced cell contraction to the same extent as the loss of ROCK1/2 or RhoC alone (Figure 4F). Our observations demonstrate that vaccinia-induced cell contraction involves RhoC-mediated activation of ROCK.

### F11-Mediated Inhibition of RhoD Promotes RhoC-Driven Cell Contraction

**Figure 5:**
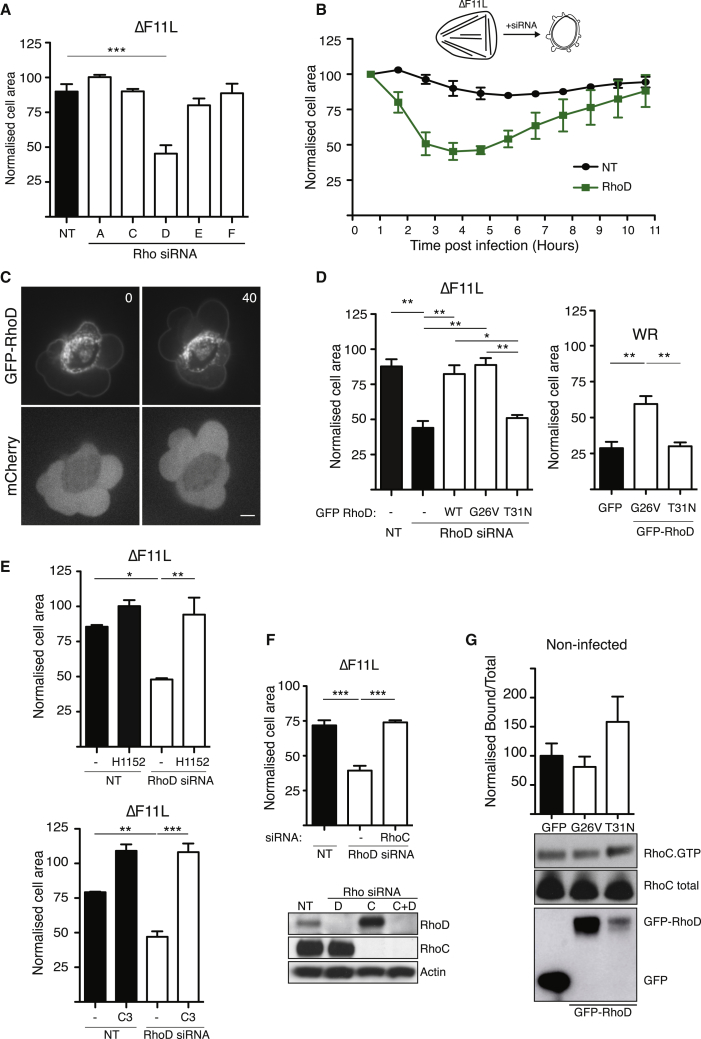
RhoD Negatively Regulates Vaccinia-Stimulated Cell Contraction (A) Quantification of the area of HeLa cells treated with the indicated siRNA for 72 hr at 3 hr 40 min post infection with the ΔF11L virus. (B) Quantification of the change HeLa cell area after treatment with RhoD siRNA and infection with the ΔF11L virus. (C) Images showing the association of GFP-RhoD with the plasma membrane in cells infected with WR for 3 hr 40 min. The time is indicated in seconds and mCherry provides a volume marker (see Movie S5). Scale bar, 5 μm. (D) Left graph shows the quantification of the area of HeLa cells, treated with RhoD siRNA and expressing GFP-tagged wild-type (WT), G26V (constitutively active), or T31N (dominant-negative) RhoD and infected with ΔF11L at 3 hr 40 min post infection. Right graph shows the area of HeLa cells expressing GFP-tagged G26V (constitutively active) or T31N (dominant-negative) RhoD and infected with WR for 3 hr 40 min. (E) The area of RhoD-depleted HeLa cells, with or without H1152 (top) or C3 (bottom) treatment, and infected with ΔF11L at 3 hr 40 min post infection. (F) The area of HeLa cells depleted of RhoD or RhoD and RhoC and infected with ΔF11L at 3 hr 40 min post infection. The immunoblot shows the efficiency of RhoD and RhoC knockdown, and actin represents a loading control. (G) The graph shows the level of GTP-bound RhoC in non-infected U-2 OS cells expressing GFP, GFP-RhoD G26V, or T31N mutant. Error bars represent SEM from four independent experiments. The immunoblot illustrates a representative assay. Error bars in cell-area graphs represent the SEM from three independent experiments, in which a total of 90 (A and B), 70 (E), and 60 (D and F) cells were analyzed. ^∗^p < 0.05, ^∗∗^p < 0.01, ^∗∗∗^p < 0.001. See also Figure S5.

It is well established that RhoGTPases frequently regulate each other (Guilluy et al., 2011). Given their interaction with F11, it is possible that RhoD, RhoE, and RhoF may have an inhibitory role in promoting vaccinia-induced cell contraction. We found that loss of RhoD but not RhoE or RhoF results in contraction of ΔF11L virus-infected cells (Figures 5A and S5A). Moreover, this contraction has temporal dynamics similar to those of WR-induced cell contraction (Figure 5B). GFP-tagged RhoD is associated with the plasma membrane of vaccinia-induced blebs (Figure 5C and Movie S5) and can also interact with GST-F11-VK in lysates from infected cells (Figure S5B). Furthermore, in vitro pull-down assays with recombinant proteins demonstrate that F11 interacts directly with RhoD (Figure S5C). We further examined the impact of expressing siRNA-resistant GFP-tagged wild-type (WT), constitutively active (G26V), or dominant-negative (T31N) RhoD on ΔF11L-infected cells treated with RhoD siRNA. GFP-tagged RhoD or its constitutively active G26V mutant suppressed the contraction of ΔF11L-infected cells induced by the depletion of endogenous RhoD (Figure 5D). In contrast, GFP-RhoD-T31N did not inhibit cell contraction. Taken together, these data suggest that RhoD and RhoC have an antagonistic role during vaccinia-mediated cell contraction. While the presence of RhoC and activity of ROCK are required for F11-induced cell contraction, RhoD must be inactive or absent. Consistent with this, GFP-RhoD-G26V, but not the dominant-negative T31N mutant, suppressed contraction of WR-infected cells (Figure 5D). To determine whether RhoD and RhoC are working in the same or parallel pathways, we investigated whether pharmacological inhibition of ROCK, or C3-mediated loss of RhoC signaling, suppresses contraction of ΔF11L-infected cells that lack RhoD. Indeed, both inhibitors blocked contraction of ΔF11L-infected cells treated with RhoD siRNA (Figure 5E). Furthermore, ΔF11L-infected cells did not contract in the absence of both RhoC and RhoD, following siRNA silencing of both proteins (Figure 5F). Curiously, loss of RhoA, which promotes the spreading of ΔF11L-infected cells, was partially able to suppress the impact of the loss of RhoD during ΔF11L infection (Figure S5D). Notwithstanding this, taken together our results demonstrate that F11 facilitates RhoC-mediated contraction of WR-infected cells by inhibiting RhoD signaling. Moreover, in non-infected cells expression of dominant-negative GFP-RhoD-T31N increases the level of GTP-bound RhoC, confirming that RhoD can inhibit RhoC activity outside the context of vaccinia infection (Figure 5G).

Our previous observations demonstrated that RhoA is inactivated by the RhoGAP activity of myosin-9A, which interacts with F11 (Handa et al., 2013). It is possible that myosin-9A bound to F11 also inhibits RhoD signaling. However, this does not appear to be the case, as WR still induces contraction of cells treated with myosin-9A siRNA (Figure S5E). Furthermore, unlike RhoD siRNA, loss of myosin-9A does not induce contraction of ΔF11L-infected cells (Figure S5E).

### RhoD Inhibits RhoC Signaling via Pak6

**Figure 6:**
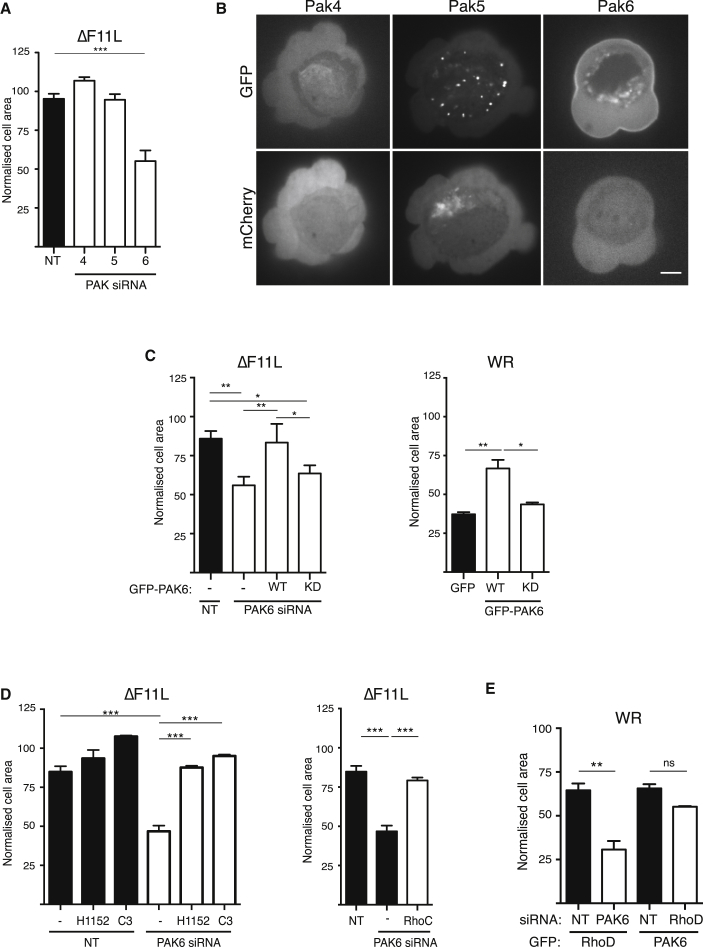
Pak6 Inhibits RhoC-Driven Cell Contraction (A) Quantification of the area of HeLa cells treated for 72 hr with the indicated siRNA and infected with the ΔF11L virus for 3 hr 40 min. (B) Images showing the subcellular localization of GFP-tagged Pak4, Pak5, and Pak6 during WR infection. Only GFP-Pak6 is associated with the plasma membrane, and mCherry provides a volume marker (see Movie S6). Scale bar, 5 μm. (C) Quantification of the area of WR- or ΔF11L-infected HeLa cells treated with Pak6 siRNA and expressing GFP-tagged WT or kinase-dead (KD) Pak6. (D) The area of Pak6 depleted HeLa cells infected with ΔF11L at 3 hr 40 min post infection and treated with H1152, C3, or RhoC siRNA. (E) The area of WR-infected HeLa cells depleted of RhoD or Pak6 and expressing GFP-tagged Pak6 or RhoD, respectively. Error bars in graphs represent the SEM from three independent experiments, in which a total of 90 (A and D) or 60 (C and E) cells were analyzed. ^∗^p < 0.05, ^∗∗^p < 0.01, ^∗∗∗^p < 0.001; ns, not significant. See also Figure S6.

There are many documented examples and different mechanisms regulating RhoGTPase crosstalk (Guilluy et al., 2011). For example, Rac antagonizes RhoA signaling via the p21-activated kinase family members, Pak1 and Pak4, which phosphorylate and inhibit a number of different Rho guanine nucleotide exchange factors (RhoGEFs) to prevent RhoA activation (Barac et al., 2004, Nimnual et al., 2003, Rosenfeldt et al., 2006). Pak5, a class II family member, is also one of a few known RhoD binding partners (Wu and Frost, 2006). Given this information, we decided to investigate whether Pak family kinases mediate the antagonistic crosstalk between RhoD and RhoC. We found that pharmacological inhibition of the class I family members Pak1–3 with IPA3 did not stimulate contraction of ΔF11L-infected cells (Figure S6A). In contrast, siRNA-mediated loss of Pak6 but not Pak4 or Pak5 results in cell contraction (Figures 6A, S6B, and S6C). Furthermore, GFP-tagged Pak6 but not Pak4 or Pak5 associates with the plasma membrane of blebs (Figure 6B and Movie S6). The ΔF11L-induced contraction of cells treated with Pak6 siRNA is specific, as it is inhibited by expression of siRNA-resistant GFP-PAK6 (Figure 6C). In contrast, the Pak6-K436A kinase-dead mutant did not suppress contraction of ΔF11L-infected cells treated with Pak6 siRNA (Figure 6C). In agreement with this, GFP-PAK6, but not its kinase-dead mutant, blocked WR-induced cell contraction (Figure 6C).

To confirm that Pak6 acts upstream of RhoC and ROCK, we examined the impact of inhibiting RhoC or ROCK with C3 and H115, respectively, in ΔF11L-infected cells depleted of Pak6. In both cases, ΔF11L-induced contraction of cells lacking Pak6 was suppressed (Figure 6D). Cells simultaneously depleted of Pak6 and RhoC also failed to contract (Figure 6D). To examine whether Pak6 and RhoD act in the same pathway to inhibit RhoC/ROCK signaling, we expressed GFP-tagged RhoD or Pak6 in cells depleted of endogenous Pak6 or RhoD, respectively. We found that the ability of GFP-RhoD to inhibit contraction of WR-infected cells depends on the presence of Pak6 (Figure 6E). In contrast, GFP-Pak6 blocks WR-induced contraction in the presence or absence of RhoD (Figure 6E). Our data demonstrate that RhoD acts through Pak6 to inhibit RhoC-mediated cell contraction.

### RhoD Interacts with Pak6 and Mediates Its Recruitment to the Plasma Membrane

**Figure 7:**
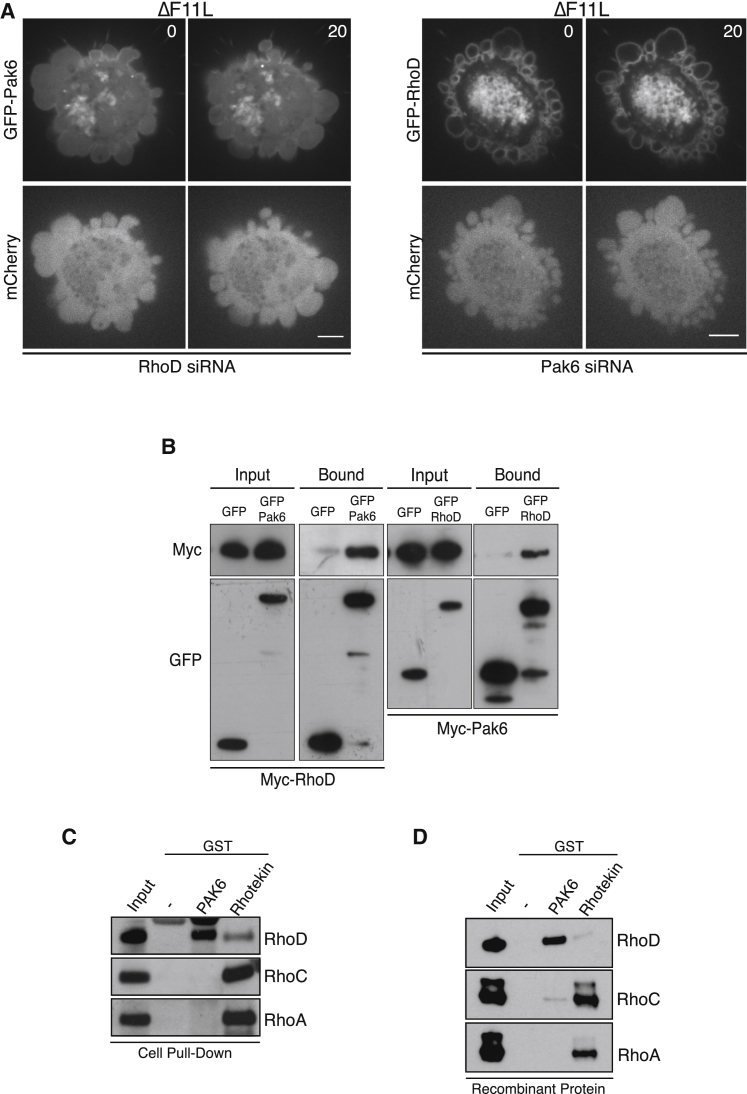
RhoD Interacts Directly with Pak6 (A) Images showing that siRNA-mediated ablation of RhoD leads to a loss of GFP-Pak6 recruitment to the plasma membrane in cells infected with ΔF11L. In contrast, loss of Pak6 does not impact on recruitment of GFP-RhoD to the plasma membrane (see Movie S7). Scale bar, 5 μm. (B) Immunoblot analysis with the indicated antibodies of a GFP-Trap pull-down on cell lysates from uninfected HeLa expressing Myc-RhoD and GFP-Pak6 or GFP-RhoD and Myc-Pak6. (C) Immunoblot of glutathione-Sepharose pull-downs on HeLa cell lysates demonstrates that GST-Pak6 interacts with RhoD, while Rhotekin preferentially associates with RhoA and RhoC. (D) Immunoblot of glutathione-Sepharose pull-downs with recombinant proteins demonstrates that GST-Pak6 interacts with RhoD but not RhoA or RhoC. See also Figure S7.

It is thought that RhoGTPases regulate class II Pak kinases by controlling their subcellular location rather than directly stimulating their kinase activity (Ha et al., 2015). We therefore examined whether RhoD is required to recruit Pak6 to the plasma membrane. We found that there was a dramatic reduction in the association of GFP-Pak6 with the plasma membrane of blebs in ΔF11L-infected cells treated with RhoD siRNA (Figure 7A and Movie S7). In contrast, loss of Pak6 had no obvious impact on the recruitment of GFP-RhoD to the plasma membrane (Figure 7A and Movie S7). These data suggest that RhoD interacts with Pak6 to recruit the kinase to the plasma membrane. Consistent with this, reciprocal pull-downs on myc- and GFP-tagged RhoD and Pak6 demonstrate that the two proteins interact with each other in uninfected cells (Figure 7B). Recombinant GST-Pak6 can also retain RhoD but not RhoC or RhoA from cell lysates (Figures 7C and S7A). Moreover, pull-down assays with recombinant proteins demonstrate that the interaction between RhoD and Pak6 is direct (Figures 7D and S7B). Our data clearly demonstrate that RhoD inhibits RhoC-ROCK-induced cell contraction via its downstream effector Pak6.

## Discussion

The organization and contraction of the cortical actin cytoskeleton plays an essential role in controlling cell shape and movement during development and tumor cell invasion (Blaser et al., 2006, Charras and Paluch, 2008, Fackler and Grosse, 2008, Friedl and Wolf, 2003, Paluch and Raz, 2013, Sahai and Marshall, 2003, Sanz-Moreno et al., 2008, Tozluoglu et al., 2013, Zatulovskiy et al., 2014). While it is well established that myosin-II drives contraction of the actin cortex, we still lack a complete understanding of the organization and composition of the cortical actin cytoskeleton, as well as the signaling networks controlling its form and function (Barry et al., 2015, Biro et al., 2013, Bovellan et al., 2014, Fritzsche et al., 2013, Fritzsche et al., 2016). One of the hallmarks of myosin-II-driven contraction of the actin cortex is the formation of blebs at the plasma membrane (Charras et al., 2008). Cell blebbing, which occurs during ameboid-based cell motility, mitotic rounding, and cytokinesis (Friedl and Wolf, 2003, Matthews et al., 2012, Sedzinski et al., 2011), is also frequently seen as part of the so-called cytopathic effect during many different viral infections (Agol, 2012). In the case of vaccinia virus, cells start to contract and bleb within a few hours of infection (Bablanian et al., 1978, Barry et al., 2015, Schepis et al., 2006, Schramm et al., 2006). We have now demonstrated that vaccinia-induced cell contraction and blebbing is dependent on the viral protein F11. The ability of F11 to induce ROCK-dependent cell contraction early during viral replication contrasts with its ability to inhibit RhoA signaling in the latter stages of infection (Arakawa et al., 2007b, Cordeiro et al., 2009, Handa et al., 2013, Valderrama et al., 2006). F11-mediated inhibition of RhoA signaling at 8 hpi stimulates infected cell migration and also facilitates the release of new viral progeny by increasing microtubule growth toward the plasma membrane and modulating the cortical actin cytoskeleton (Arakawa et al., 2007a, Arakawa et al., 2007b, Cordeiro et al., 2009, Handa et al., 2013, Morales et al., 2008, Valderrama et al., 2006).

Considering the ability of RhoA to bind F11, it was surprising that F11-induced cell contraction depends on RhoC and not RhoA, as both GTPases have been shown to bind and activate ROCK to induce actin stress fiber formation and myosin-II contraction (Leung et al., 1996, Ridley, 2013). Historically it has been difficult to assign individual effects or cellular responses to RhoA and RhoC, in part because, until relatively recently, most studies tended to focus only on RhoA. Furthermore, expression of constitutively active or dominant-negative RhoA mutants impacts on the activity of both GTPases, as does the inhibitory effect of C3 toxin (Aktories, 2011). More recently, the use of RNAi-based approaches has begun to uncover distinct functions for RhoA and RhoC (Bravo-Cordero et al., 2011, Bravo-Cordero et al., 2013, Korkina et al., 2013, Sahai and Marshall, 2003, Vega et al., 2011). However, it is striking that the evidence for RhoA driving cell contraction and blebbing is largely based on the use of reagents that do not fully discriminate between RhoA and RhoC (Charras et al., 2006, Costigliola et al., 2010, Gutjahr et al., 2005, Sahai and Marshall, 2003, Sanz-Moreno et al., 2008). Our observations demonstrate that RhoC plays the greater role in stimulating ROCK-mediated myosin-II contraction of the actin cortex during vaccinia infection. This conclusion has important implications for a variety of cellular processes involving cell contraction and blebbing that have previously only been assigned to RhoA. It is also striking that there is more evidence suggesting that RhoC is more important than RhoA in promoting tumor cell invasion and metastasis (Clark et al., 2000, Dietrich et al., 2009, Hakem et al., 2005, Kitzing et al., 2010, Leung et al., 1996, Ridley, 2013, Ruth et al., 2006, Sahai and Marshall, 2002, Simpson et al., 2004).

F11 interacts directly with RhoA using a motif that is similar to that of the Rho-binding site in ROCK (Cordeiro et al., 2009, Valderrama et al., 2006). It is likely that F11 will also bind directly to RhoC, as its effector binding switchI/II regions are essentially identical to those in RhoA (Ridley, 2013, Wheeler and Ridley, 2004). Our in vitro pull-down assays with recombinant protein reveal that RhoC interacts much less efficiently with F11 than with RhoA (Figure S5C). The reason for this dramatic difference is not immediately obvious. However, we found that F11 regulates RhoC-mediated cell contraction by inhibiting RhoD signaling. RhoD is emerging as a RhoGTPase family member with diverse functions including regulation of centrosome duplication, cell-cycle progression, actin structures, and membrane trafficking (Gasman et al., 2003, Koizumi et al., 2012, Kyrkou et al., 2013, Nehru et al., 2013). Previous analysis demonstrates that overexpression of constitutively active RhoD disrupts focal adhesions and stress fibers, and also suppresses the ability of active RhoA to promote actin assembly (Tsubakimoto et al., 1999). These overexpression data led the authors to suggest that RhoD antagonizes RhoA signaling (Tsubakimoto et al., 1999). Our data now clearly demonstrate that RhoD antagonizes the function of RhoC, which represents another example of RhoGTPase crosstalk, whereby the action of one GTPase regulates the activity of another. GTPase crosstalk can regulate RhoGEFs and RhoGAPs, GTPase stability, and downstream signaling (reviewed in Guilluy et al., 2011). One well-documented example of GTPase crosstalk is the inhibition of RhoA by Rac. Following Rac activation, the downstream effectors Pak1 and Pak4 phosphorylate p115-RhoGEF, GEF-H1, PDZ-RhoGEF, and Net1 (Alberts et al., 2005, Barac et al., 2004, Rosenfeldt et al., 2006, Zenke et al., 2004). Phosphorylation of these RhoGEFs inhibits their exchange activity or alters their stability and/or localization, leading to a loss of downstream RhoA activation. There remains the possibility that Pak1 and Pak4 can also control RhoC-mediated cell contraction, as some of these RhoGEFs also regulate the activity of RhoC (Jaiswal et al., 2011, Ren et al., 1998). This possibility, together with the observation that RhoD interacts with Pak5 (Wu and Frost, 2006), led us to investigate whether Pak kinases were involved in vaccinia-induced cell contraction. We found that only depletion of Pak6 stimulated RhoC/ROCK-dependent contraction of ΔF11L virus-infected cells. Conversely, Pak6 overexpression suppressed contraction of WR-infected cells. Furthermore, RhoD suppressed WR-induced cell contraction through Pak6, suggesting that Pak6 is a RhoD effector. Consistent with this notion, we found that RhoD can interact directly with Pak6 and is responsible for recruitment of the kinase to the plasma membrane.

Our data are in agreement with previous observations suggesting that RhoGTPases principally regulate class II Pak family members by controlling their subcellular location rather than directly stimulating their kinase activity (Ha et al., 2015). Nevertheless, our results clearly show that the kinase activity of Pak6 is required to inhibit RhoC-induced cell contraction. It is possible that Pak6 may directly phosphorylate RhoC to regulate its activity. Seven of the 22 RhoGTPases, including RhoA, have a serine residue between their phospholipid-binding polybasic region and the CAAX motif that can be phosphorylated (Wheeler and Ridley, 2004). Phosphorylation of this serine weakens the association of the RhoGTPase with the membrane and also increases its affinity for Rho GDP-dissociation inhibitor (RhoGDI), which extracts the GTPase from membrane (Boulter et al., 2010, Ellerbroek et al., 2003, Garcia-Mata et al., 2011). However, RhoC does not have an equivalent serine residue between its polybasic region and CAAX motif, so is unlikely to be regulated by this mechanism. Furthermore, our pull-down assays demonstrate that Pak6 does not interact with RhoC. Given this finding it may be that, as with Rac-mediated inhibition of RhoA, Pak6 suppresses the activity of RhoC by phosphorylating one or more RhoGEF. This raises the question of how specificity of a RhoGEF(s) for RhoC over RhoA is achieved. The RhoGEF binding switch I regions of RhoA, B, and C are almost identical, with only a single change for RhoB (Q29E) or RhoC (V43I) (Wheeler and Ridley, 2004). Structural analysis of RhoA in complex with the LARG, PDZ-RhoGEF, or p63RhoGEF reveals that Val43 participates in van der Waals interactions at the RhoGTPase:GEF interface (Chen et al., 2010, Kristelly et al., 2004, Lutz et al., 2007, Oleksy et al., 2006). A bulkier isoleucine residue at this point could sterically block the interaction between the RhoGTPase and its GEF. Indeed, the GEF XPLN interacts with RhoA and RhoB but not RhoC, the distinguishing feature being the isoleucine residue at position 43 in RhoC (Arthur et al., 2002, Sloan et al., 2012). Conversely, it is also conceivable that RhoD/Pak6 can activate a GAP capable of downregulating RhoC signaling. We previously found that RhoA is inactivated by the GAP activity of myosin-9A, in an F11-dependent manner in late stages of infection (Handa et al., 2013). However, we now found that in early stages of infection there is no role for myosin-9A, as WR still induces cell contraction and those infected with ΔF11L remain spread when myosin-9A is depleted (Figure S5E). These data also suggest that myosin-9A has no GAP activity toward RhoD.

From our studies it is clear that early during vaccinia infection there must be an additional stimulus activating RhoC, as ΔF11L virus-infected cells depleted of RhoD or Pak6 contract in an RhoC-dependent fashion. This is consistent with our hypothesis that in ΔF11L-infected cells RhoD recruits Pak6 to the plasma membrane where it can locally antagonize RhoC signaling to ROCK to inhibit cell contraction. Moreover, the activation of RhoC by an additional viral protein within the first few hours of infection might explain why F11 promotes very different phenotypes at early and late time points during infection. It is also possible that the selectivity of RhoC during vaccinia-induced contraction is actually dependent on this unknown stimulus, leaving the possibility open that Pak6 suppresses both RhoA and RhoC signaling. The task ahead is to understand how vaccinia activates RhoC and to use vaccinia-induced cell contraction as an assay to uncover the basis for Pak6-mediated inhibition of RhoC signaling.

## STAR★Methods

### Key Resources Table

**Table:** 

REAGENT or RESOURCE	SOURCE	IDENTIFIER
**Antibodies**		

Mouse Monoclonal anti-β-Actin (AC-74)	Sigma-Aldrich	Cat#A2228; RRID: AB_476697
Rabbit Anti-Glutathione-S-Transferase (GST) antibody	Sigma-Aldrich	Cat#G7781; RRID: AB_259965
Mouse Monoclonal Anti-polyHistidine antibody (clone HIS-1)	Sigma-Aldrich	Cat#H1029; RRID: AB_260015
Mouse Monoclonal Anti-c-Myc antibody (clone 9E10)	Cancer Research UK	N/A
Mouse Monoclonal Anti-Mcm7 antibody (clone DCS-141)	Sigma-Aldrich	Cat#M7931; RRID: AB_260666
Mouse Monoclonal Anti-alpha-Tubulin Antibody (clone B-5-1-2)	Sigma-Aldrich	Cat#T6074; RRID: AB_477582
Rabbit Anti-Zip Kinase antibody	Sigma-Aldrich	Cat#Z0134; RRID: AB_261899
Rabbit monoclonal MYPT1 (D6C1)	Cell Signaling Technology	Cat#8574S; RRID: AB_10998518
Rabbit Phospho-MYPT1 (Thr696) Antibody	Cell Signaling Technology	Cat#5163S; RRID: AB_10691830)
Rabbit Phospho-Myosin Light Chain 2 (Ser19) Antibody	Cell Signaling Technology	Cat#3671S; RRID: AB_330248
Rabbit monoclonal RhoA (67B9) antibody	Cell Signaling Technology	Cat#2117S; RRID: AB_10693922
Rabbit Monoclonal Anti-RhoC (Clone D40E4)	Cell Signaling Technology	Cat#3430S; RRID: AB_2179246
Rabbit Monoclonal ROCK1 (C8F7)	Cell Signaling Technology	Cat#4035S; RRID: AB_2238679
Rabbit Monoclonal ROCK2 (D1B1)	Cell Signaling Technology	Cat#9029S;RRID: AB_11127802
Rabbit Polyclonal Anti-Human PAK5	ProSci	Cat#3075; RRID: AB_735027
Rabbit Polyclonal PAK6 antibody	GeneTex	Cat#GTX127915; RRID: N/A
Rabbit polyclonal RHOD antibody	Anbobio	Cat#C18411; RRID: N/A
Rabbit polyclonal RHOD antibody	OriGene	Cat#TA312722; RRID: N/A
Mouse Monoclonal anti-GFP (clone 3E1)	Cancer Research UK	N/A
Mouse Monoclonal Anti-GRB2 Antibody (clone 81)	BD Transduction	Cat#610112, RRID: AB_397518
Rabbit anti-A36	Röttger et al., 1999	N/A
Rabbit anti-F11-N	Handa et al., 2013	N/A
Rabbit anti-F11-C	This paper	N/A
Rabbit anti-MRCKα	Wilkinson et al., 2005	N/A
Peroxidase-AffiniPure Goat Anti-Rabbit IgG antibody	Jackson ImmunoResearch Labs	Cat# 111-035-003; RRID: AB_2313567
Peroxidase-AffiniPure Goat Anti-Mouse IgG antibody	Jackson ImmunoResearch Labs	Cat#115-035-003; RRID: AB_10015289
FITC-Donkey anti-Rabbit IgG	Jackson ImmunoResearch Labs	Cat#711-095-152; RRID:AB_2315776
Texas Red-X Phalloidin	Molecular Probes	Cat# T7471

**Bacterial and Virus Strains**		

Vaccinia virus Western Reserve	Cudmore et al., 1995	N/A
Vaccinia virus Western Reserve ΔF11L	Cordeiro et al., 2009	N/A
Vaccinia virus Western Reserve F11-VK	Cordeiro et al., 2009	N/A
Vaccinia virus Western Reserve GFP-F11	This paper	N/A
Escherichia coli BL21-CodonPlus (DE3)-RIL	Agilent Technologies	Cat# 230245

**Chemicals**, **Peptides**, **and Recombinant Proteins**		

Rho inhibitor (C3)	Cytoskeleton Inc.	Cat#CT04
ROCK inhibitor Y27632	Sigma-Aldrich	Cat#Y0503
ROCK inhibitor H1152	Tocris Bioscience	Cat#2414
ROCK inhibitor GSK429286A	Tocris Bioscience	Cat#3726
Myosin Light Chain Kinase (MLCK) inhibitor ML7	Sigma-Aldrich	Cat#I2764
Myosin Light Chain Kinase (MLCK) inhibitor ML9	Sigma-Aldrich	Cat# C1172
Cyclohexamide (CHX)	Sigma-Aldrich	Cat#C7698
Group I p21-activated kinase (PAK) inhibitor IPA-3	Tocris Bioscience	Cat#3622
EDTA-free Protease Inhibitor Cocktail	Roche	Cat#05056489001
PhosSTOP Phosphatase inhibitor tablets	Roche	Cat#04906837001
PDIKLDAVLDRDGNFRPADC (F11 residues 101-120)	Handa et al., 2013	N/A
CGGNFITKEIKNRDK (F11 residues 323-334)	This paper	N/A
HiPerFect Transfection Reagent	Qiagen	Cat#301707
FuGENE 6 Transfection Reagent	Promega	Cat#E2691
GST-F11	Valderrama et al., 2006	N/A
GST-Pak6	This paper	N/A
GST-Rhotekin (residues 7-89)	Millipore	Cat# 14-383
His-RhoA	This paper	N/A
His-RhoB	This paper	N/A
His-RhoC	This paper	N/A
His-RhoD	This paper	N/A
His-RhoE	This paper	N/A
His-RhoF	This paper	N/A
His-Rac1	This paper	N/A

**Critical Commercial Assays**		

GFP-Trap_A	ChromoTek	Cat#GTA-20
Rho Assay Reagent (Rhotekin RBD, agarose)	Millipore	Cat#14-383
Glutathione Sepharose 4B	Amersham	Cat#17-0756-01
Ni-NTA resin	Qiagen	Cat#30230
RNeasy Mini kit	Qiagen	Cat#74104
SuperScript II reverse transcriptase	Invitrogen	Cat#18064-014
ECL Western Blot detection reagent	Amersham	Cat#RPN2106

**Experimental Models**: **Cell Lines**		

Human: HeLa cells	ATCC	Cat#CCL-2
Human: U-2 OS cells	The Francis Crick Institute Cell Services	N/A

**Oligonucleotides**		

siRNA sequences	This paper, see Table S1	N/A

**Recombinant DNA**		

pCB6-GFP	Handa et al., 2013	N/A
pCB6-Myc	Handa et al., 2013	N/A
pMW172-GST	Boeda et al., 2007	N/A
pMW172-His	Boeda et al., 2007	N/A
pEL-GFP	Frischknecht et al., 1999	N/A
pEL-GST	Frischknecht et al., 1999	N/A
pLVX MLC2-RFP	Barry et al., 2015	N/A
pEL-GST-F11L	Valderrama et al., 2006	N/A
pMW-GST-F11L	Valderrama et al., 2006	N/A
pEL-GFP-RhoA	Valderrama et al., 2006	N/A
pMW-His-RhoA	Valderrama et al., 2006	N/A
pEL-GFP-RhoB	This paper	N/A
pMW-His-RhoB	This paper	N/A
pEL-GFP-RhoC	This paper	N/A
pMW-His-RhoC	This paper	N/A
pCB6 GFP-RhoC WT	This paper	N/A
pCB6 GFP-RhoC T19N	This paper	N/A
pEL-GFP-RhoD	This paper	N/A
pMW-His-RhoD	This paper	N/A
pCB6 GFP-RhoD WT	This paper	N/A
pCB6 GFP-RhoD T31N	This paper	N/A
pCB6 GFP-RhoD G26V	This paper	N/A
pCB6 Myc-RhoD	This paper	N/A
pEL-GFP-RhoE	This paper	N/A
pMW-His-RhoE	This paper	N/A
pEL-GFP-RhoF	This paper	N/A
pMW-His-RhoF	This paper	N/A
pEL-GFP-Rac1	This paper	N/A
pMW-His-Rac1	This paper	N/A
pCB6-GFP-Pak4	This paper	N/A
pCB6-GFP-Pak5	This paper	N/A
pCB6-GFP-Pak6	This paper	N/A
pCB6-GFP-Pak6 KD	This paper	N/A
pCB6-Myc-Pak6	This paper	N/A
pLVX-GFP	Barry et al., 2015	N/A
pLVX-mCherry	Barry et al., 2015	N/A

**Software and Algorithms**		

MetaMorph Microscopy Automation and Image Analysis Software	Molecular Devices	https://www.moleculardevices.com/systems/metamorph-research-imaging
ImageJ	National Institutes of Health	https://imagej.nih.gov/ij/
SlideBook	Intelligent Imaging Innovations	https://www.intelligent-imaging.com/slidebook

**Other**		

NuPAGE 4-12% Bis-Tris Protein Gels, 1.0 mm, 10-well	Invitrogen	Cat#NP0321BOX
NuPAGE 3-8% Tris-Acetate Protein Gels, 1.0 mm, 10-well	Invitrogen	Cat#EA0375BOX

### Contact for Reagent and Resource Sharing

Further information and requests for resources and reagents should be directed to and will be fulfilled by the Lead Contact, Michael Way (michael.way@crick.ac.uk).

### Experimental Model and Subject Details

#### Cell Culture

HeLa cells were cultured in Modified Eagle Medium (MEM) supplemented with 10% SFB, penicillin (100u/ml) and streptomycin (100μg/ml). U-2 OS cells were cultured in Dulbecco’s Modified Eagle Medium (DMEM) supplemented with 10% SFB, penicillin (100u/ml) and streptomycin (100μg/ml). HeLa and U2-OS cells were authenticated by STR profiling and are both negative for mycoplasma.

#### Viruses and Infections

The wild-type virus used in this work is the Western Reserve strain (WR) (Cudmore et al., 1995). The recombinant viruses ΔF11L or F11-VK have been previously described (Cordeiro et al., 2009). The virus encoding GFP-F11, with a GGRGG linker between the GFP and N-terminus of F11 was generated by homologous recombination between a targeting vector encoding F12-GFP-F11 and the ΔF12L virus (Dodding et al., 2009, Zhang et al., 2000, Rietdorf et al., 2001). The F12-GFP-F11 targeting vector was transfected into HeLa cells 3 hours after infection with the ΔF12L virus, at a multiplicity of infection of 0.1 plaque-forming units. Two days later, cells were harvested and ruptured by freeze thawing. The cell lysate was then used to infect confluent BSC-1 cell monolayers, which were overlaid with 0.9% agarose 2 h after infection. Four days later, individual plaques were picked based on the rescue of the ΔF12L small plaque phenotype as well as expression of GFP. The virus in the isolated plaques was then amplified by re-infecting HeLa cells for 48 h. A further 4 rounds of plaque purification were performed until the recombinant GFP-F11 virus was clonal. The fidelity of the recombinant virus was confirmed by sequencing. HeLa or U-2 OS cells were infected with the WR strain of Vaccinia or its recombinant F11L derivatives at a multiplicity of infection (MOI) of 5 in serum free MEM. After 20 minutes the media was replaced for MEM +10%FBS.

### Method Details

#### Plasmids

GFP, GST, Myc and His-tagged Pak4, Pak5, Pak6, RhoA to RhoF and Rac1 expression constructs were generated as required by cloning their respective ORFs into the Not1-EcoR1 sites of CB6-GFP or CB6-Myc a CMV based mammalian expression vector (Handa et al., 2013); pMW172-GST or pMW172-His an *E. coli* T7 based expression vector (Boeda et al., 2007) and pEL-GFP or pEL-GST a vaccinia expression vector (Frischknecht et al., 1999). Human RhoB and RhoF (Rif) as well as mouse RhoD templates were a kind gift of Dr. Harry Mellor (Bristol University, UK). Human RhoC and RhoE were a kind gift of Dr. Erik Sahai (The Francis Crick Institute, UK). Human PAK 4, 5 and 6 synthetic gene sequences were bought from GeneArt (Invitrogen). GST-F11 in pMW172 and pEL expression vectors have been described (Valderrama et al., 2006). The pLVX CMV based mammalian expression clones for MLC2-RFP, GFP and mCherry have been described (Barry et al., 2015). Point mutations were introduced into wild-type clones as required using the QuikChange site directed mutagenesis kit (Stratagene).

#### Drug Treatments

The cell permeable Rho inhibitor (C3) (Cytoskeleton Inc. USA) was added to cells at a final concentration of 0.5μg/ml one hour prior to infection. The ROCK inhibitors 10μM Y27632 (Sigma Aldrich), 5μM H1152 or 5μM GSK429286A and the Group I p21-activated kinase (PAK) inhibitor 10μM IPA-3 (Tocris Bioscience) were added to cells 30 minutes before infection. The Myosin Light Chain Kinase (MLCK) inhibitors 20μM ML7 or ML9 (Sigma Aldrich) were added to cells 15 minutes after viral infection in the presence or absence of Cyclohexamide (CHX) at a final concentration of 20μg/ml. ML7 or ML9 were maintained throughout the experiment after CHX washout at 2hpi.

#### siRNA and DNA Transfections

Dharmacon pools of four siRNA oligo duplexes against Pak4 (MU-003615-00), Pak5 (MU-003973-02), Pak6 (MU-004338-02), RhoA (MU-003860-03), RhoC (MU-008555-01), RhoD (MU-008940-0), RhoE (MU-007794-02), Rif (MU-008316-00) and DAPK3/ZIPK (MU-004947-00) were used for knockdown. In addition, two individual siRNA oligo duplexes against RhoD (oligo #1 D-008940-01 and oligo #2 D-008940-02) and Pak6 (oligo #1 D-004338-05 and oligo #2 D-004338-06) were also used. Individual oligos were also used to deplete Myosin-9A (D-006539-01 and D-006539-03), ROCK1 (D-003536-05) and ROCK2 (D-004610-05) as previously described (Pinner and Sahai, 2008). MRCKα and β were depleted using siRNA MRCK1 (CGAGAAGACTTTGAAATAA) targeting both isoforms and MRCK2 targeting MRCKα (AAGAATATCTGCTGTGTTT) and MRCKβ (GAAGAATACTGAACGAATT) as described in Wilkinson et al., 2005. HeLa cells were transfected with 20nM of siRNA using the HiPerFect fast-forward protocol (Qiagen). After three days, the cells were processed for immunoblotting, RT-qPCR or infected with vaccinia virus for live cell imaging. Sixteen hours prior to infection, HeLa cells were transfected with the indicated expression vectors using Fugene6 (Roche).

#### Reverse Transcription Quantitative PCR (RT-qPCR)

Total RNA was extracted from HeLa cell lysates using the RNeasy Mini kit (Qiagen) according to the manufacturer’s protocol. RNA was converted to cDNA using SuperScript II reverse transcriptase (Applied Biosystems). cDNA was amplified by quantitative PCR using Power SYBR Green reagents and 7500 Fast Real-Time PCR System (Applied Biosystems). The level of mRNA of the gene of interest (GOI) was normalized to GAPDH mRNA and normalized GOI mRNA in knockdown samples was compared to AllStar control sample (NT) using the comparative Ct method.

#### Purification of Recombinant Proteins

The expression vector pMW-GST-F11 was transformed into E. coli BL21-Codon Plus (DE3)-RIL (Agilent Technologies) and expressed for 16h at 30°C. Cells were collected by centrifugation, resuspended in bacterial lysis buffer [50mM Tris-HCl pH8.0, 150mM NaCl, 1mM EDTA, 0,1% Triton-X, 25% sucrose, protease inhibitor cocktail (Roche)] and lysed by sonication. The soluble fraction was collected by centrifugation and incubated with Glutathione Sepharose 4 Fast Flow beads (Amersham) for 1h at 4°C. GST-F11 coupled beads were collected by centrifugation, washed three times and resuspended in GST- wash/storage buffer [PBSA, 250mM NaCl, 0,1% Triton-X, 10% glycerol].

The expression vector pMW-GST-Pak6 was transformed into E. coli BL21-Codon Plus (DE3)-RIL (Agilent Technologies) and expressed for 16h at 30°C. Cells were collected by centrifugation, resuspended in GST-Pak6 lysis/wash buffer [PBS, 1% Triton-X, 50mM NaF, 1mM Na_3_VO_4,_ 1mM PMSF, protease inhibitor cocktail (Roche, UK)] and lysed by sonication. The soluble fraction was collected by centrifugation and incubated with Glutathione Sepharose 4B beads (Amersham) for 1h at 4°C. GST-Pak6 coupled beads were collected by centrifugation, washed three times with GST-Pak6 lysis/wash buffer and resuspended in GST-Pak6 Storage buffer [50% glycerol, 20mM Tris-HCl pH7.6, 100mM NaCl and 1mM DTT].

All pMW-His Rho clones and Rac1 were transformed into E. coli BL21-Codon Plus (DE3)-RIL (Agilent Technologies) and expressed for 16h at 30°C. Cells were collected by centrifugation, resuspended in bacterial lysis buffer [50mM Tris-HCl pH8.0, 150mM NaCl, 0,1% Triton-X, 1mM EDTA, 25% sucrose, protease inhibitor cocktail (Roche, UK)] and lysed by sonication. The soluble fraction was collected by centrifugation and imidazole at pH8.0 added to a final concentration of 50mM. The soluble fraction was then incubated with Ni-NTA resin (Qiagen) for 1h at 4°C. His-tagged protein coupled beads were collected by centrifugation, washed three times with Ni wash buffer [PBSA, 250mM NaCl, 0,1% Triton-X, 50mM imidazole and 10mM β-mercaptoethanol]. Purified His proteins were eluted from Ni beads in Ni wash buffer plus 250mM imidazole for 30 min at 4°C.

#### Pull Downs Assays

##### GFP-Trap Pull Down Assays

For GFP-Trap pull down assays U2-OS cells were transfected with combinations of CB6 expression vectors encoding Myc- and GFP-tagged RhoD and Pak6 according to the manufacturer’s protocol (Fugene6, Roche). Sixteen hours later, the transfected cells were lysed in 50mM Tris-HCl pH7.5, 300mM NaCl, 10% glycerol, 1% Triton and protease inhibitor cocktail (Roche). Lysates were incubated with GFP-Trap beads (ChromoTek) for 1hour at 4°C, collected by centrifugation, washed three times with lysis buffer and resuspended in 2 x SDS loading buffer.

##### GST Pull Down Assays

Vaccinia virus infected HeLa cells were co-transfected with pEL-GST-F11 or pEL-GST-F11-VK and pEL-GFP-tagged RhoGTPases 4 hours post infection prior to cell lysis 8 hours later. Cells were lysed in Mg^2+^ Lysis/Wash buffer [25mM Hepes pH7.5, 150mM NaCl, 1% NP-40, 10mM MgCl_2_, 1mM EDTA, 2%glycerol] supplemented with 25mM NaF, 20mM PMSF, 20mM Orthovanadate and protease inhibitor cocktail (Roche). Lysates were cleared by centrifugation and were incubated with Glutathione Sepharose 4 beads (Amersham) for 60min at 4°C, collected by centrifugation, washed three times with Mg^2+^ Lysis/Wash buffer and resuspended in 2 x SDS loading buffer.

Pulldowns on HeLa lysates with recombinant GST-Pak6 or GST-Rhotekin (Millipore) were performed as previously described (Fram et al., 2014). Briefly, cells were lysed in NP-40 lysis buffer [0,5% NP-40, 50mM Tris-HCl pH7.6, 150mM NaCl, 0.1mM EDTA, 50mM NaF, 1mM DTT and protease and phosphatase inhibitor cocktails (Roche, UK)]. Lysates were pre-cleared by incubation with GST-coupled Glutathione Sepharose 4 Fast Flow beads (Amersham) for 30min at 4°C, then incubated with GST-Pak6 beads for 90min at 4°C, collected by centrifugation, washed three times with lysis buffer and resuspended in 2 x SDS loading buffer.

Recombinant pulldown assays with recombinant His-tagged Rho proteins loaded with GTPγS and GST, GST-F11, GST-Pak6 or GST-Rhotekin were performed as previously described (Cordeiro et al., 2009, Handa et al., 2013). Briefly, His-tagged proteins were loaded with 10mM GTPγS for 30 minutes at 30°C after addition of 10mM EDTA. Then, MgCl_2_ at a final concentration of 10mM was added to the loaded His-tagged proteins, which were then incubated with GST-tagged proteins for 1h at 4°C. Beads were collected by centrifugation, washed three times with GST (when GST-F11 was used as resin) or GST-Pak6 (when GST-Pak6 was used as resin) wash buffer and resuspended in 2 x SDS loading buffer.

##### Rho Activity Assays

Rhotekin pull down assays were performed on uninfected U-2 OS expressing GFP, GFP-RhoD G26V or T31N that were serum starved for 24 hours and stimulated with 20% FBS for 5 minutes. Cells were lysed in Mg^2+^ Lysis/Wash buffer [25mM Hepes pH7.5, 150mM NaCl, 1% NP-40, 10mM MgCl_2_, 1mM EDTA, 2%glycerol]. Lysates were pre-cleared by centrifugation and were incubated with GST-Rhotekin beads (Millipore) for 45min at 4°C, collected by centrifugation, washed three times with Mg^2+^ Lysis/Wash buffer and resuspended in 2 x SDS loading buffer.

#### Immunoblotting

Proteins were loaded on NuPAGE™ 3-8% Tris-Acetate (for visualizing MCM-7, MRCK and MYPT) Protein Gels or NuPAGE 4-12% Bis-Tris (all other proteins) Protein Gels (Invitrogen) and run at 150V for 90 minutes, after which time they were transferred to nitrocellulose membranes using an iBlot dry blotting system (Invitrogen). Blots were then washed 3x 5 minutes in PBS, following by blocking in 5% milk in PBS-T (PBS plus 0,1% Tween). Primary antibodies were then incubated in 5% BSA in PBS-T overnight at 4°C. Blots were washed 3x 5 minutes in PBS-T, following by incubation with secondary antibodies for 1 hour in 5% milk in PBS-T. Blots were washed 3x 5 minutes in PBS-T and proteins were visualized using ECL western detection reagent (Amersham) and imaged with X-ray films.

#### Live Cell Imaging

##### Cell Contraction Assays

Cells were seeded onto fibronectin-coated 12 well plates, transfected (if required) with 20nM of siRNA against MRCK, ROCK, ZipK, Rho and PAK proteins using the HiPerFect fast-forward protocol (Qiagen) three days before infection, and (if required) with the indicated expression vectors (pCB6GFP-RhoC, pCB6GFP-RhoD, pCB6GFP-PAK6 and their mutants variants) using Fugene6 (Roche) sixteen hours prior to infection. When indicated, cells were treated with chemical inhibitors prior to infection as described in details in the drug treatment section. HeLa cells were then infected with the WR strain of vaccinia or its recombinant F11L derivatives at a multiplicity of infection (MOI) of 5 in serum free MEM. After 20 minutes the media was replaced for MEM +10%FBS supplemented with 40mM HEPES and the indicated drugs and 40 minutes post infection images were acquired using a Plan-Apochromat 10x/0.25 Ph1 lens and a Photometrics Cool Snap HQ cooled CCD camera on a Zeiss Axiovert 200 Microscope every 5 minutes for 10 hours. The system was controlled by MetaMorph software version 6.3r7 (Molecular Devices). The average area of infected cells was determined at 1-hour intervals from the start of image acquisition using MetaMorph or ImageJ. The average cell area at each time point was normalized to the original mean and in all experiments is represented as the percentage of the original cell area. All cell quantifications where GFP-tagged proteins were expressed were only performed on cells with a GFP signal that was collected in parallel to the phase images.

##### Localization of GFP/RFP-tagged Proteins in Blebs

Localization of GFP-tagged proteins in blebs was performed as previously described (Barry et al., 2015). Cells were seeded onto fibronectin-coated 35-mm MatTek dishes, transfected using FuGENE 6 (Promega) with pLVX-GFP or pLVX-mCherry (as cytoplasmic markers) and with the specific pCB6-tagged proteins constructs, and cultured overnight. Cells were then infected with a WR or ΔF11L strain of vaccinia virus at a multiplicity of infection (MOI) of 5 in serum free MEM. After 20 minutes the media was replaced with phenol red–free MEM with 10%FBS and live-cell imaging begun 3 h after infection using a Plan Apochromat 63×/1.40 NA oil objective (Carl Zeiss) in a temperature-controlled chamber at 37°C. Images were captured on a Evolve 512 camera (Photometrics) mounted on an inverted AxioObserver.Z1 microscope (Carl Zeiss) as part of a custom-built spinning-disc confocal system (Intelligent Imaging Innovations). All hardware was controlled with SlideBook software (Intelligent Imaging Innovations). Images were collected at 1 second intervals.

#### Confocal Microscopy

Cells were seeded onto fibronectin-coated coverslips and cultured overnight. When indicated, cells were treated with ROCK inhibitor H1152 prior to infection as described in details at the drug treatment section. HeLa cells were then infected with a WR or ΔF11L strain of vaccinia virus at a multiplicity of infection (MOI) of 5 in serum free MEM. After 20 minutes the media was replaced for MEM +10%FBS supplemented with H1152 when indicated. At 3 hours and 40 minutes post infection, cells were fixed with 4% PFA for 10 minutes at room temperature and washed three times with PBS. Cells were then permeabilized with 0,1% Triton X-100 in PBS for three minutes and washed three times with PBS. Cells were incubated in blocking buffer (BB) [10mM MES pH6.1, 150mM NaCl, 5mM EGTA, 5mM MgCl_2_, 5mM glucose] for 10 minutes and afterwards with pMLC-S19 primary antibody diluted in BB for 1 hour at room temperature. Cells were then washed three times with PBS and incubated with FITC-anti-Rabbit and Texas-Red Phalloidin diluted in BB for 30 min at room temperature. Cells were then washed three times with PBS, rinsed with distilled water, mounted in microscopy slides using Mowiol and dried. Images were acquired using a Zeiss LSM 780 Confocal Microscope.

### Quantification and Statistical Analysis

In all graphs the data is the mean value from at least three independent experiments in which 60-100 cells were analysed and the error bars represent the standard error of the mean (S.E.M.). Statistical analysis was determined using Prism 5.0 (GraphPad Software, CA). A Student’s T-test was used to compare two data sets. When more than two data sets were analyzed a One Way ANOVA test was performed followed by Turkey post test to compare all pairs of samples. A P value of >0.05 is not considered statistically significant. ^∗^ indicates P<0.05, ^∗∗^ indicates P<0.01 and ^∗∗∗^ indicates P<0.001.

## Author Contributions

C.H.D., F.L., and M.W. designed the study and wrote the manuscript. C.H.D., F.L., and J.V.C. performed and analyzed the experiments. Y.A. and F.V. performed initial experiments leading to this study. Y.H. generated the recombinant GFP-F11 virus. All authors commented on the manuscript text.

## Supplemental Information

Document S1. Figures S1–S7 and Table S1

Movie S1. Vaccinia F11 Promotes Cell Contraction Early during Infection, Related to Figures 1 and S1

Movie S2. F11 Is Recruited to the Plasma Membrane, Related to Figures 1 and S1

Movie S3. MLC2 Is Recruited to the Plasma Membrane of Retracting Blebs, Related to Figures 2 and S2

Movie S4. RhoC Is Recruited to the Plasma Membrane, Related to Figures 4 and S4

Movie S5. RhoD Is Recruited to the Plasma Membrane, Related to Figures 5 and S5

Movie S6. Pak6 but Not Pak4 or Pak5 Is Recruited to the Plasma Membrane, Related to Figures 6 and S6

Movie S7. Pak6 Recruitment to the Plasma Membrane Is Dependent on RhoD, Related to Figures 7 and S7

Document S2. Article plus Supplemental Information
