# Supplementary material for: RhoD Inhibits RhoC-ROCK-Dependent Cell Contraction via PAK6
Source: Dev Cell. 2017 May 8;41(3):315–329.e7. doi: 10.1016/j.devcel.2017.04.010 (PMC5425256; doi:10.1016/j.devcel.2017.04.010)
Supplement: Document S1. Figures S1–S7 and Table S1 [file mmc1.pdf]

Developmental Cell, Volume *41*

Supplemental Information

RhoD Inhibits RhoC-ROCK-Dependent Cell Contraction via PAK6

Charlotte H. Durkin, Flavia Leite, João V. Cordeiro, Yutaka Handa, Yoshiki Arakawa, Ferran Valderrama, and Michael Way

Supplemental Information

RhoD inhibits RhoC-ROCK dependent cell contraction via PAK6

Charlotte H. Durkin, Flavia Leite, João V. Cordeiro, Yutaka Handa, Yoshiki Arakawa, Ferran Valderrama and Michael Way

INVENTORY OF SUPPLEMENTAL INFORMATION

Supplemental figures

Figure S1. WR but not MVA stimulates cell contraction (Related to Figure 1)
(A) Phase contrast images showing the morphology of U-2 OS cells at the indicated times after infection with WR or the ΔF11L virus. The immunoblot reveals the level of F11 expression U-2 OS cells at the indicated times after infection with WR. A36 and Grb2 represent viral and HeLa cell loading controls respectively. The graph shows the quantification of the average area of U-2 OS cells infected with WR (black) or ΔF11L (red) over 11 hours. **(B)** Phase contrast images showing the morphology of HeLa cells at the indicated time points after infection with WR or MVA. Quantification of the average area of HeLa cells infected with WR (black) or MVA (blue) over 11 hours. All error bars represent the S.E.M from three independent experiments, in which a total of 60 cells were analyzed.

Figure S2
WR induces redistribution of MLC2 but does not impact on its phosphorylation (Related to Figure 2)
(A) Immunoblot with the indicated antibodies reveals the ROCK inhibitors GSK49286A (G), H1152 (H) and Y27632 (Y) inhibit phosphorylation of MLC2 and MYPT1. The images show representative confocal images of actin cytoskeleton and the distribution of phosphorylated MLC2 in HeLa cells infected with WR, in the presence or absence of H1152, and the ΔF11L virus. **(B)** Immunoblot analysis of the level of A36 in WR infected cells reveals that treatment with ROCK inhibitors does not inhibit viral infection. Grb2 represents a cell loading control. **(C)** The immunoblot shows the efficiency of siRNA knockdown of ROCK1 and ROCK2 in HeLa cells after 72 hours of RNAi treatment. Actin represents a cell loading control. **(D)** Immunoblot analysis of the level of A36 in WR infected cells reveals that siRNA knockdown of ROCK1 and ROCK2 does not inhibit viral infection. Grb2 represents a cell loading control. **(E)** Immunoblot shows the level of phosphorylated MLC2 in non-infected (MOCK) and WR or ΔF11L infected cells at the indicated time points. **(F)** Images showing the association of MLC2-RFP with the plasma membrane of blebs in cells infected with WR for 3:40 hours. The time is indicated in seconds and GFP provides a volume marker (See Movie 2). Scale Bar = 5µm

Figure S3 C3 does not inhibit vaccinia infection (Related to Figure 3)
(A) Immunoblot analysis of the level of A36 expression at the indicated time points reveals that C3 does not impair infection or viral protein expression.

Figure S4 Analysis of siRNA mediated RhoGTPase knockdown efficiency (Related to Figure 4)

(A) The immunoblot reveals the level of RhoA and/or RhoC in HeLa cells 72 hours after siRNA treatment. NT is the non-targetting AllStar siRNA control and tubulin represents a cell loading control. **(B)** The Immunoblot shows the level of RhoD in HeLa cells 72 hours after siRNA treatment. The graphs show RT-qPCR analysis of the percentage of remaining RhoD, RhoE or RhoF mRNA following siRNA treatment relative to the NT non-targetting AllStar control (NT) siRNA and error bars represent S.E.M. from two independent experiments.

Figure S5 RhoD siRNA promotes contraction of ΔF11L infected cells (Related to Figure 5)

(A) Representative phase contrast images and quantification of the area of HeLa cells treated with the indicated RhoD siRNA at 3:40hpi with the ΔF11L virus. The immunoblot shows the efficiency of RhoD knockdown with actin as a loading control. **(B)** Immunoblot analysis of glutathione sepharose pulldowns on lysates from infected HeLa cells reveals that GST-tagged F11 and its VK mutant, deficient in RhoA binding, can interact with GFP-RhoD. **(C)** Immunoblot of glutathione sepharose pulldowns with recombinant proteins demonstrates that GST-F11 can interact well with His tagged RhoA, RhoD, RhoE and RhoF but not RhoB, RhoC or Rac1. **(D)** The area of HeLa cells depleted of RhoA or RhoA and RhoD and infected with ΔF11L at 3:40hpi. NT represents the non-targetting AllStar control siRNA. The immunoblot shows the efficiency of RhoA and RhoD knockdown and actin represents a loading control. **(E)** The area of HeLa cells depleted of Myosin-9A and infected with WR or ΔF11L at 3:40hpi. NT represents the non-targetting AllStar control siRNA. All error bars in the graphs represent the S.E.M from three independent experiments, in which a total of 60 cells were analysed and a P value of <0.05 and <0.01 is indicated by * and ** respectively and ns indicates not statistically significant.

Figure S6 siRNA mediated knockdown efficiency of Pak6 (Related to Figure 6)

(A) Quantification of the area of HeLa cells treated with DMSO (control) or IPA-3 (Pak1-3 inhibitor) 3:40 hours after infection with the ΔF11L virus. **(B)** Quantification of the level of Pak4, Pak5 or Pak6 mRNA following siRNA treatment for 72 hours. NT represents the non-targetting AllStar control siRNA. The immunoblots show the level of Pak5 and Pak6 after siRNA treatment. **(C)** The left graph shows the quantification of the level of Pak6 mRNA in non-infected HeLa cells following siRNA treatment with the indicated oligos for 72 hours. The right graph shows the quantification of the area of Pak6 depleted HeLa cells infected with ΔF11L at 3:40hpi. NT represents the non-targetting AllStar control siRNA. All error bars in the cell area graphs represent the S.E.M from three independent experiments, in which a total of 90 (A) or 60 (C) cells were analysed. NS indicates not statistically significant, while a P value of <0.05 and <0.01 is indicated by * and ** respectively.

Figure S7 Purified proteins used for pulldown assays (Related to Figure 7)

(A) Coomassie stained SDS-PAGE gel showing the purified recombinant GST, GST-Pak6 and GST-Rhotekin bound to glutathione sepharose that was used for the pull down assays on cell lysates shown in Figure 7C. **(B)** The upper panel shows a coomassie stained SDS-PAGE gel showing the purified recombinant GST, GST-Pak6 and GST-Rhotekin bound to glutathione sepharose that was used for the pull down assays on His-tagged RhoA, RhoC and RhoD in Figure 7D. The lower panel shows a coomassie stained gel of the purified His-tagged RhoA, RhoC and RhoD loaded with GTPγS.

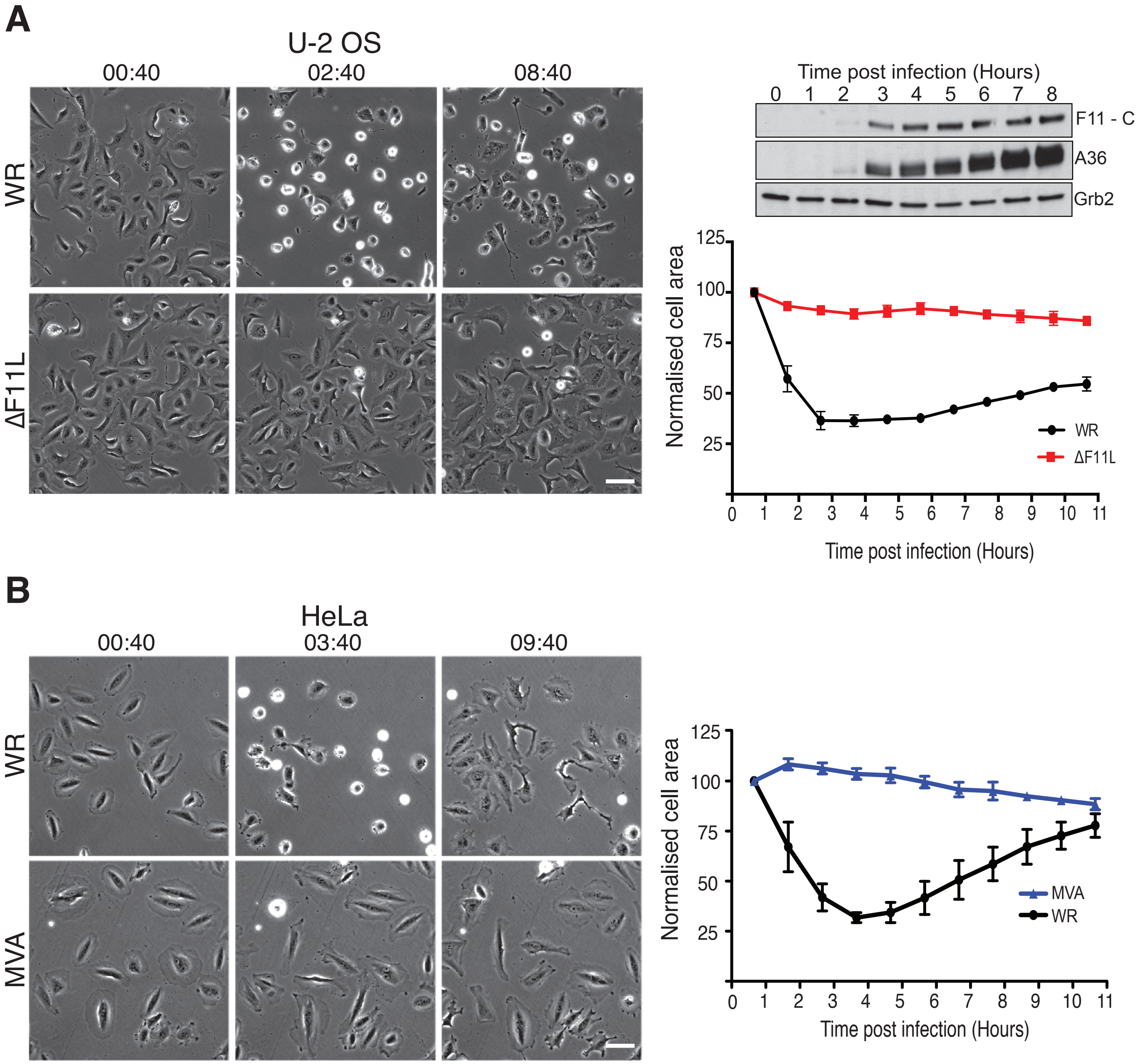

Figure S1 related to Fig 1 Durkin et al.

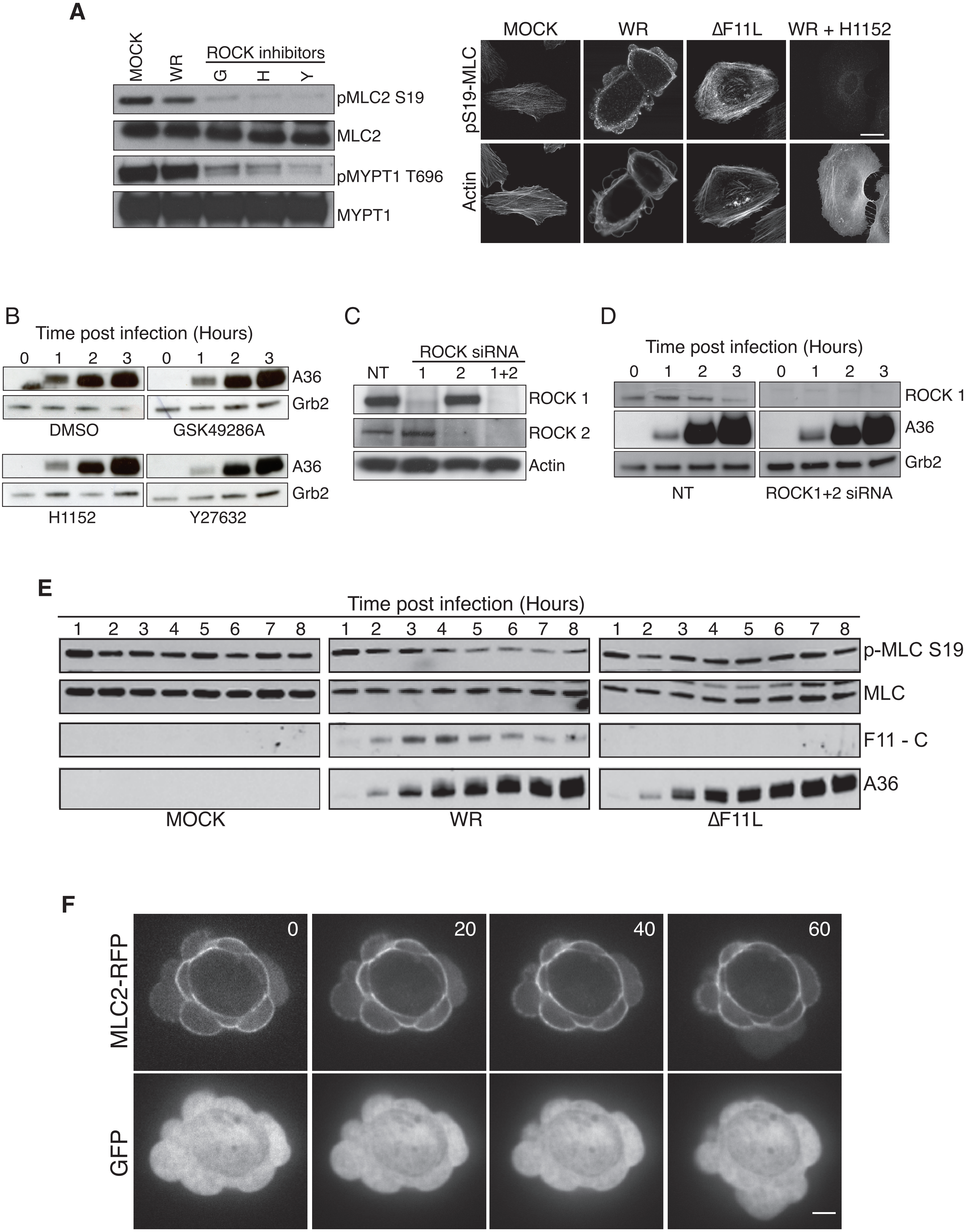

Figure S2 related to Fig 2 Durkin et al.

A

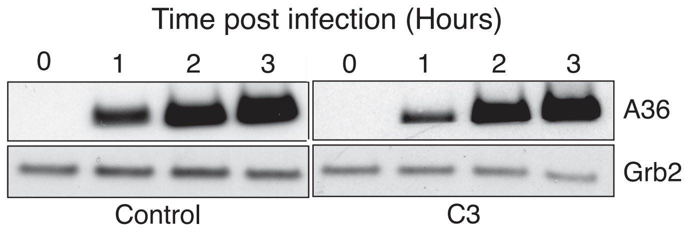

Figure S3 related to Fig 3 Durkin et al.

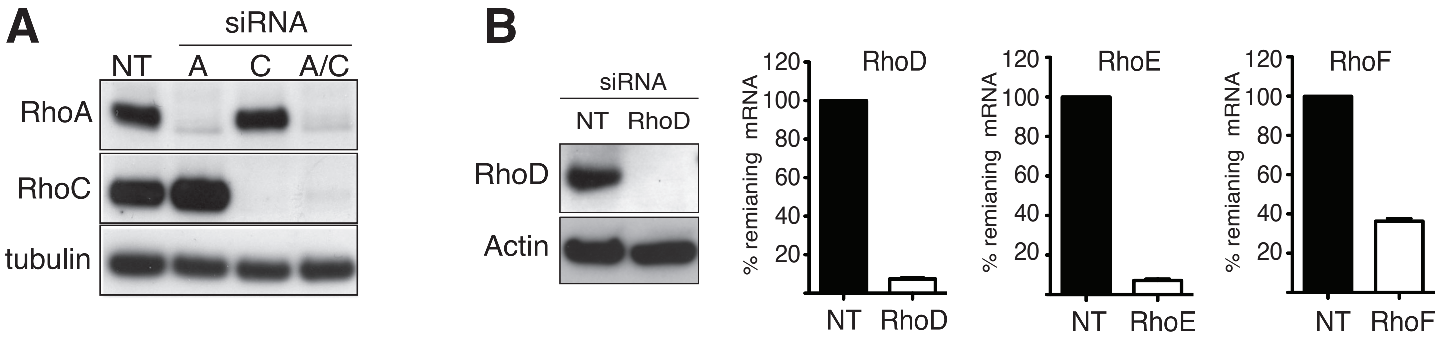

Figure S4 related to Fig 4 Durkin et al.

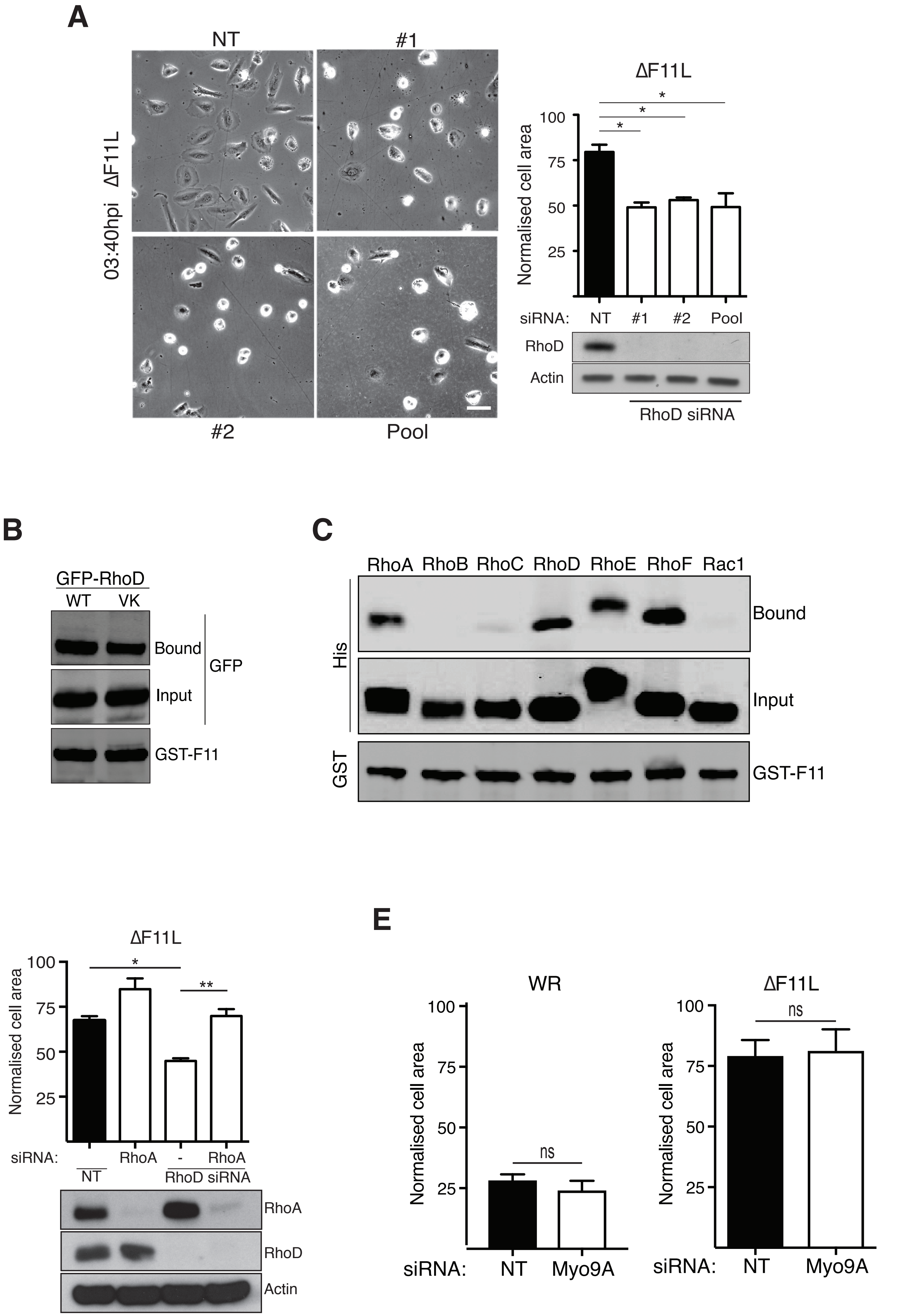

Figure S5 related to Fig 5 Durkin et al.

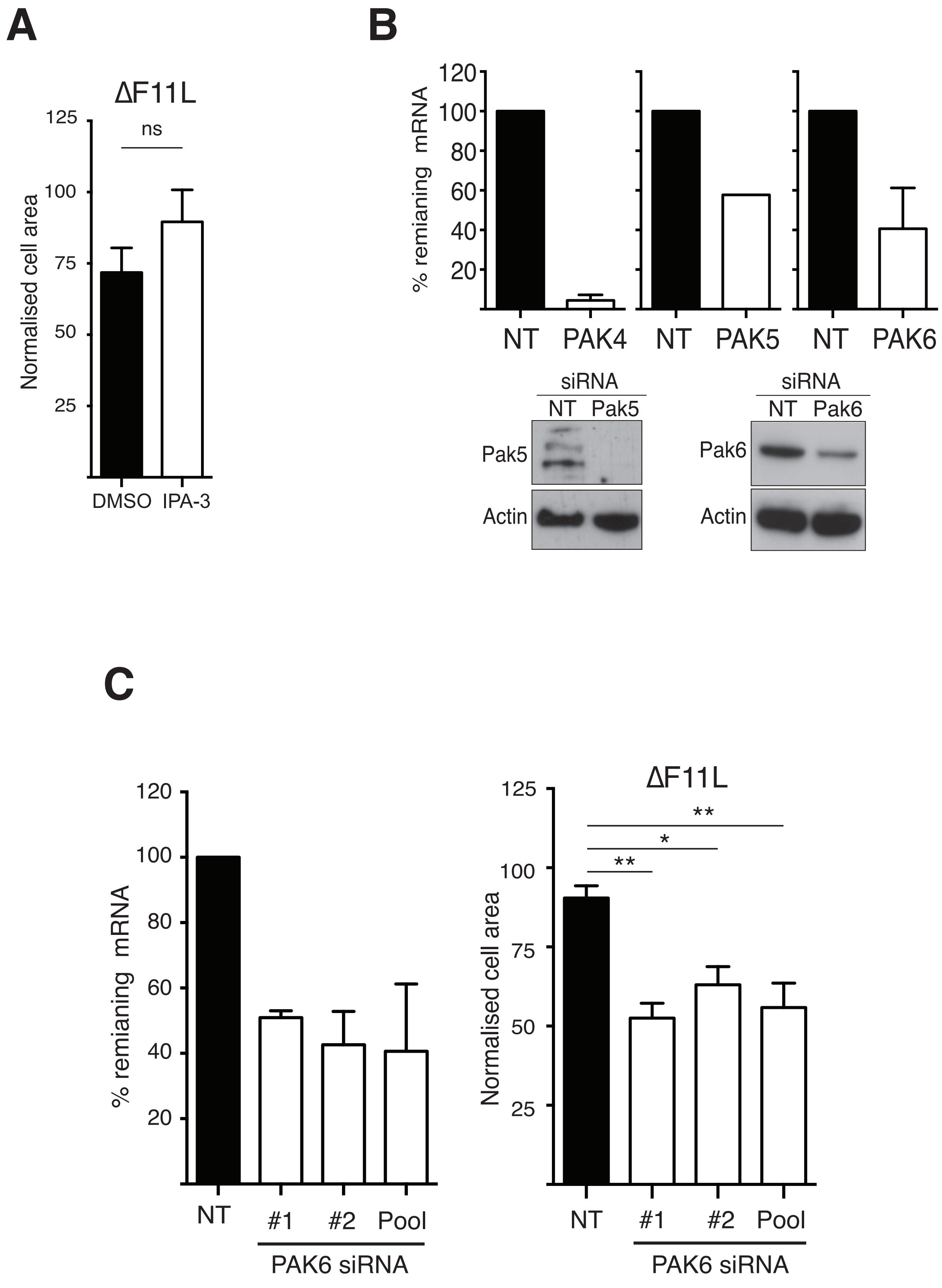

Figure S6 related to Fig 6 Durkin et al.

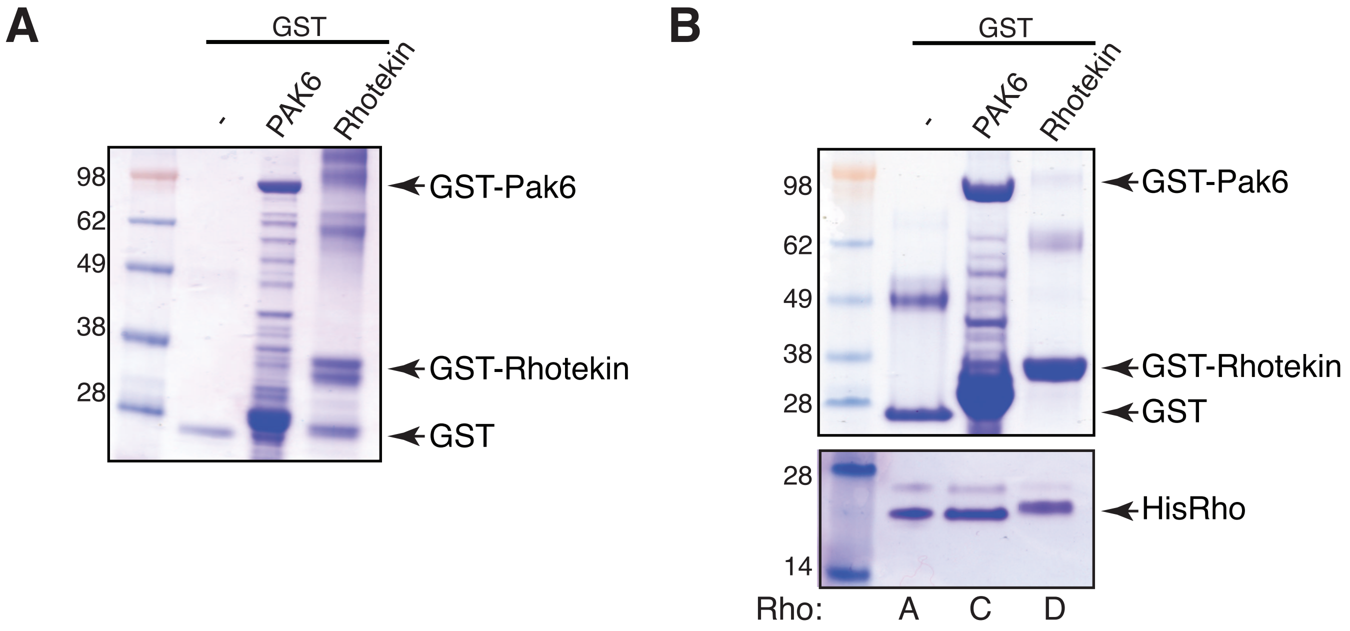

Figure S7 related to Fig 7 Durkin et al.

Table S1. siRNA sequences, related to STAR Methods

siGENOME Set of 4 Upgrade siRNA Human PAK4	GE Dharmacon	Cat#MU-003615-02
siGENOME Set of 4 Upgrade siRNA Human PAK5	GE Dharmacon	Cat# MU-003973-02
siGENOME Set of 4 Upgrade siRNA Human PAK6	GE Dharmacon	Cat# MU-004338-02
siGENOME Set of 4 Upgrade siRNA Human RHOA	GE Dharmacon	Cat# MU-003860-03
siGENOME Set of 4 Upgrade siRNA Human RHOC	GE Dharmacon	Cat#MU-008555-01
siGENOME Set of 4 Upgrade siRNA Human RHOD	GE Dharmacon	Cat#MU-008940-00
siGENOME Set of 4 Upgrade siRNA Human RHOE	GE Dharmacon	Cat# MU-007794-02
siGENOME Set of 4 Upgrade siRNA Human RHOF	GE Dharmacon	Cat# MU-008316-00
siGENOME Set of 4 Upgrade siRNA Human ZIPK	GE Dharmacon	Cat# MU-004947-00
siGENOME Human RHOD siRNA - oligo #1	GE Dharmacon	Cat# D-008940-01
siGENOME Human RHOD siRNA - oligo #2	GE Dharmacon	Cat# D-008940-02
siGENOME Human PAK6 siRNA - oligo #1	GE Dharmacon	Cat# D-004338-05
siGENOME Human PAK6 siRNA - oligo #2	GE Dharmacon	Cat# D-004338-06
siGENOME Human Myosin-9A siRNA - oligo #1	GE Dharmacon	Cat# D-006539-01
siGENOME Human Myosin-9A siRNA - oligo #2	GE Dharmacon	Cat# D-006539-03
siGENOME Human ROCK1 siRNA - oligo #1	GE Dharmacon	Cat# D-003536-05
siGENOME Human ROCK2 siRNA - oligo #1	GE Dharmacon	Cat# D-004610-05
MRCK1 siRNA targeting sequence: Human MRCKα and MRCKβ: ACACAGUACUCAGUUGAUA	Wilkinson et al., 2005	N/A
MRCK2 siRNA targeting sequences: Human MRCKα (AAGAATATCTGCTGTGTTT) and Human MRCKβ (GAAGAATACTGAACGAATT)	Wilkinson et al., 2005	N/A
