# Supplementary figures and images for: RhoD Inhibits RhoC-ROCK-Dependent Cell Contraction via PAK6
Source: Dev Cell. 2017 May 8;41(3):315–329.e7. doi: 10.1016/j.devcel.2017.04.010 (PMC5425256; doi:10.1016/j.devcel.2017.04.010)

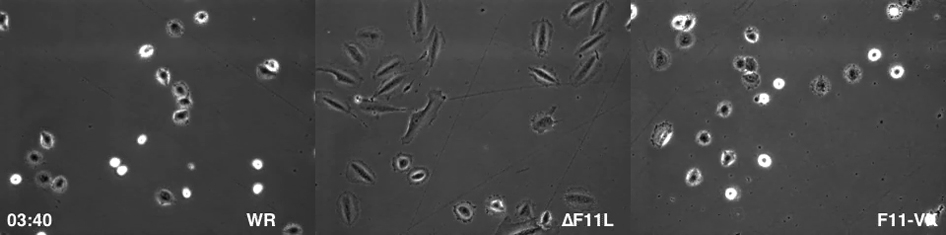

Supplement: Movie S1. Vaccinia F11 Promotes Cell Contraction Early during Infection, Related to Figures 1 and S1 [file mmc2.jpg]

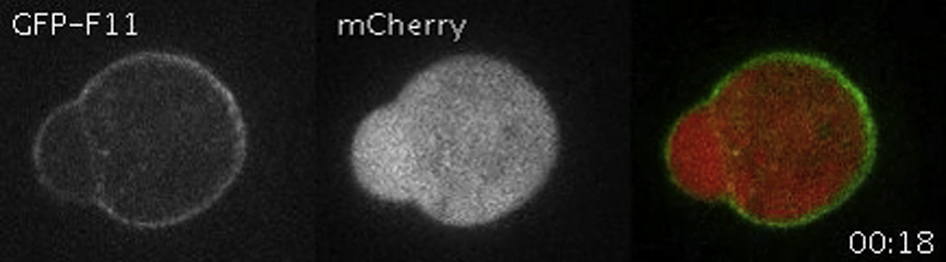

Supplement: Movie S2. F11 Is Recruited to the Plasma Membrane, Related to Figures 1 and S1 [file mmc3.jpg]

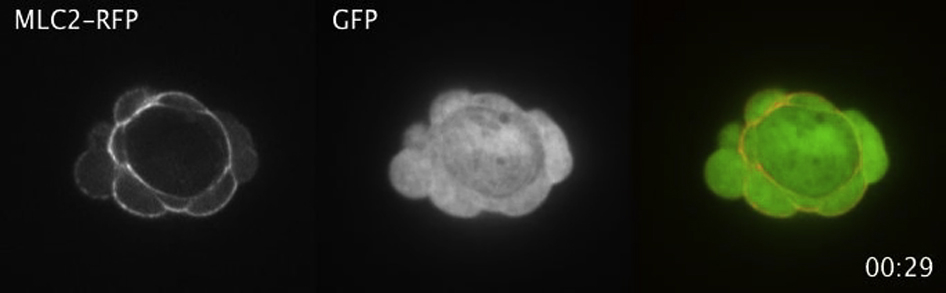

Supplement: Movie S3. MLC2 Is Recruited to the Plasma Membrane of Retracting Blebs, Related to Figures 2 and S2 [file mmc4.jpg]

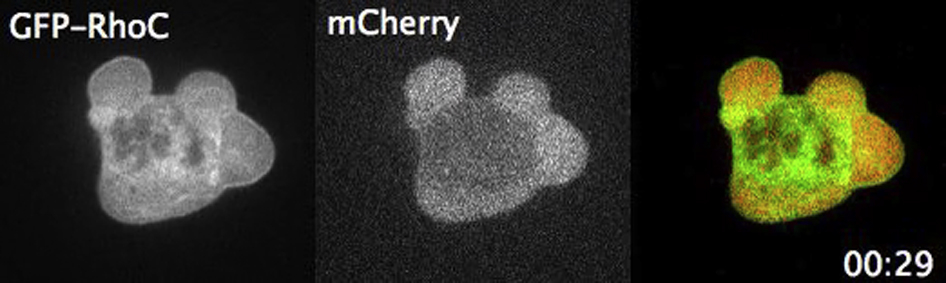

Supplement: Movie S4. RhoC Is Recruited to the Plasma Membrane, Related to Figures 4 and S4 [file mmc5.jpg]

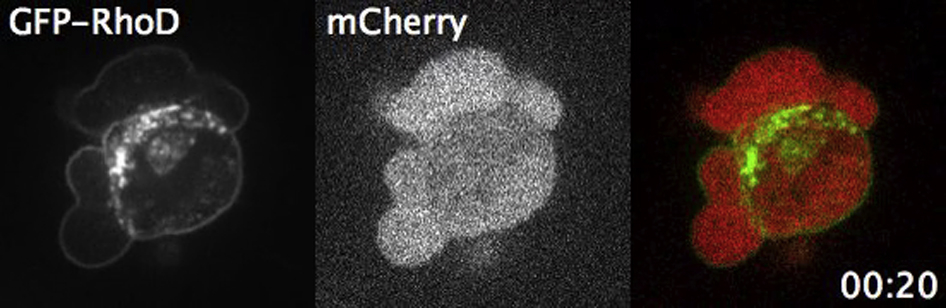

Supplement: Movie S5. RhoD Is Recruited to the Plasma Membrane, Related to Figures 5 and S5 [file mmc6.jpg]

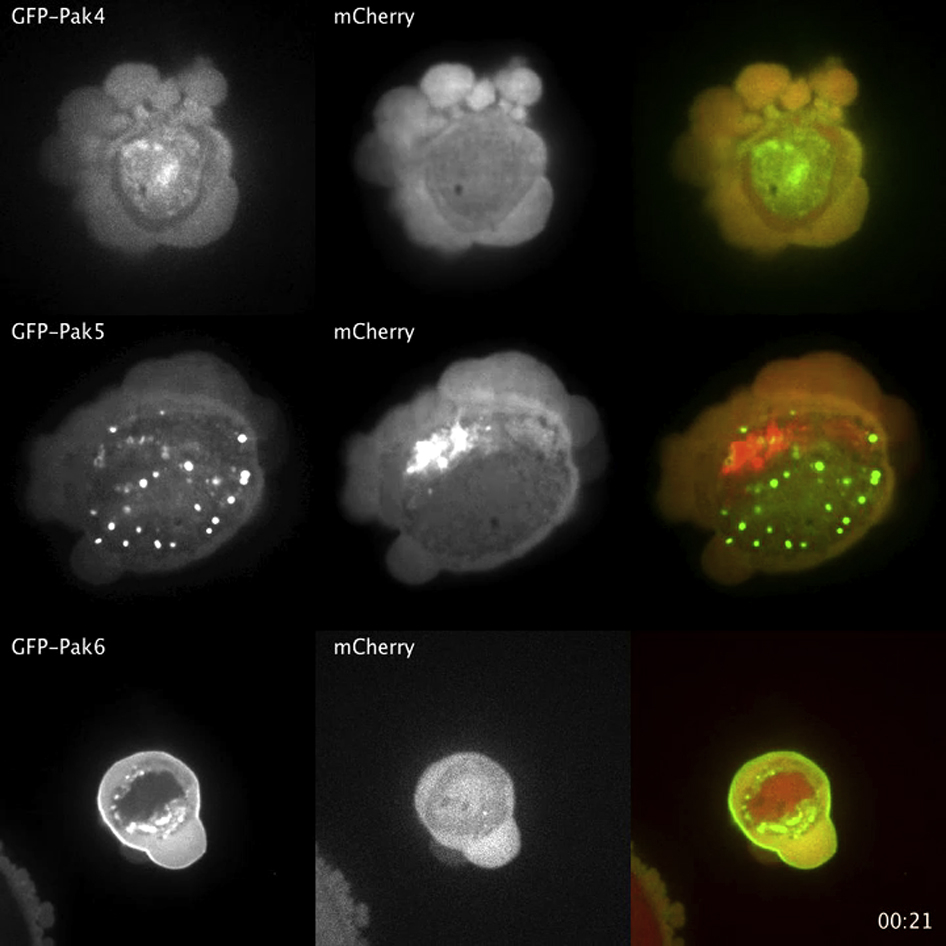

Supplement: Movie S6. Pak6 but Not Pak4 or Pak5 Is Recruited to the Plasma Membrane, Related to Figures 6 and S6 [file mmc7.jpg]

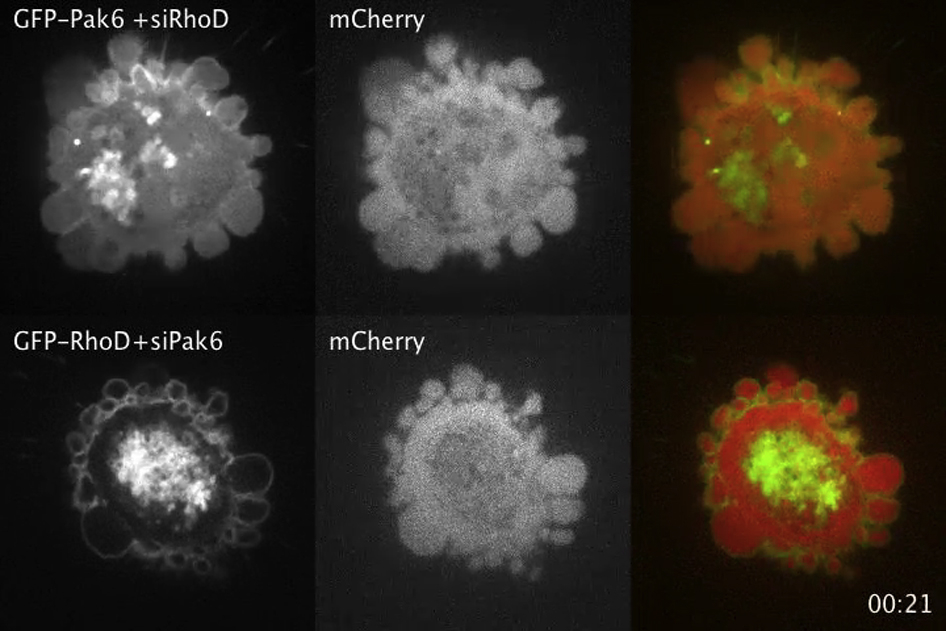

Supplement: Movie S7. Pak6 Recruitment to the Plasma Membrane Is Dependent on RhoD, Related to Figures 7 and S7 [file mmc8.jpg]
